# Gut microbiota and atopic dermatitis: a two-sample Mendelian randomization study

**DOI:** 10.3389/fmed.2023.1174331

**Published:** 2023-06-22

**Authors:** Yan Xue, Linzhu Zhang, Yajun Chen, Han Wang, Jiang Xie

**Affiliations:** ^1^The Third People’s Hospital of Chengdu, Clinical College of Southwest Jiao Tong University, Chengdu, China; ^2^School of Basic Medical Sciences, Chengdu University of Traditional Chinese Medicine, Chengdu, China; ^3^North Sichuan Medical College, Nanchong, Sichuan, China

**Keywords:** gut flora, gut microbiota, atopic dermatitis, Mendelian randomization, single nucleotide polymorphisms

## Abstract

**Background:**

Accumulating evidence suggests that alterations in gut microbiota composition and diversity are associated with Atopic dermatitis (AD). But until now, the causal association between them has been unclear.

**Methods:**

We employed a two-sample Mendelian Randomization (MR) study to estimate the potential causality of gut microbiota on AD risk. The summary statistics related to the gut microbiota were obtained from a large-scale genome-wide genotype and 16S fecal microbiome dataset from 18,340 individuals (24 cohorts) analyzed by the MiBioGen Consortium, comprising 211 gut microbiota. AD data were also derived from strictly defined AD data collected by FinnGen biobank analysis, which included 218,467 European ancestors (5,321 AD patients and 213,146 controls). The inverse variance weighted method (IVW), weighted median (WME), and MR-Egger were used to determine the changes of AD pathogenic bacterial taxa, followed by sensitivity analysis including horizontal pleiotropy analysis, Cochran’s Q test, and the leave-one-out method to assess the reliability of the results. In addition, MR Steiger’s test was used to test the suppositional relationship between exposure and outcome.

**Results:**

A total of 2,289 SNPs (*p* < 1 × 10^−5^) were included, including 5 taxa and 17 bacterial characteristics (1 phylum, 3 classes, 1 order, 4 families, and 8 genera), after excluding the IVs with linkage disequilibrium (LD). Combining the analysis of the results of the IVW models, there were 6 biological taxa (2 families, and 4 genera) of the intestinal flora positively associated with the risk of AD and 7 biological taxa (1 phylum, 2 classes, 1 order, 1 family, and 2 genera) of the intestinal flora negatively associated. The IVW analysis results showed that Tenericutes, Mollicutes, Clostridia, Bifidobacteriaceae, Bifidobacteriales, *Bifidobacterium*, and Christensenellaceae R 7 group were negatively correlated with the risk of AD, while Clostridiaceae 1, Bacteroidaceae, Bacteroides, Anaerotruncus, the unknown genus, and Lachnospiraceae UCG001 showed the opposite trend. And the results of the sensitivity analysis were robust. MR Steiger’s test showed a potential causal relationship between the above intestinal flora and AD, but not vice versa.

**Conclusion:**

The present MR analysis genetically suggests a causal relationship between changes in the abundance of the gut microbiota and AD risk, thus not only providing support for gut microecological therapy of AD but also laying the groundwork for further exploration of the mechanisms by which the gut microbiota contributes to the pathogenesis of AD.

## 1. Introduction

Atopic dermatitis (AD) (1, 2), also known as atopic eczema or hereditary allergic eczema, is a chronic inflammatory skin lesion characterized by dry, itchy skin as well as recurrent episodes. AD can occur at all ages (3), with the majority having an onset in infancy; some severe impairments can extend chronically into adulthood. Davies et al. (4) outlined 15 studies published in 2015 and found that the global prevalence of AD in childhood was 7.89% and that people with AD were four times more likely to have allergic rhinitis and asthma than the normal population. The WHO global burden of disease data suggests that at least 230 million people worldwide suffer from AD and that AD has jumped to become the fourth leading cause of non-fatal disease (5). Almost 20% of children in Western countries suffer from AD (6).

Multiple factors, including genetics, the environment, and immunity, influence AD (7), and its etiology and pathogenesis are unknown. Many researchers found that changes in intestinal flora abundance may be related to the occurrence and development of AD, but the specific relationship between the two remains unclear (8, 9). Early hypotheses were used to posit that excessive hygiene disrupts skin surface flora and thereby renders probiotics less protective for the host immune system. And then, with the rise of the “gut-skin axis “and the “gut-brain axis” (10, 11), accumulating evidence has proved that altered gut microbiota composition and diversity can affect skin immunity and metabolisms (12, 13), such as promoting cytokine responses and regulatory T cell differentiation (14). Besides, it also plays a key role in immune activation and tolerance. Short-chain fatty acids (SCFAs, such as propionate, and butyrate) bind to short-chain fatty receptors 2 (FFA2) and 3(FFA3) to induce Treg cells to regulate immune balance (15), and amino acid metabolites (tryptophan, kynurenic acid, etc.) can also play an anti-inflammatory role by binding to G-protein receptors (such as GPR142, GPR35) (16, 17).

In 2018, a systematic review of the literature on gut microbiota versus AD by Petersen (18) found that nearly half of the findings agreed that gut microbiota diversity, as well as the colonization of specific flora (such as Clostridium species, *Lactobacillus paracasei*, and *Bifidobacterium*), was inversely associated with AD risk, while the remaining studies suggested no clear association between the two based on PRISMA guidelines. A previous birth cohort study with a 16S ribosomal RNA gene sequencing approach revealed reduced gut microbiota diversity in AD patients (19). The scholar Abrahamsson also reached a similar conclusion (20) and showed that Bacteroidetes decreased at both phylum and genus levels in 1-month-old children with AD. Moreover, a study published by Nowrouzian and colleagues also found that intestinal colonization with *Staphylococcus aureus* (*S. aureus*) strains carrying a certain combination of superantigen and adhesin genes was not only negatively associated with the subsequent development of atopic eczema but also, to some extent, able to promote the maturation of the infant immune system (21). However, the potential causal relationship between gut microbiota and the risk of AD has not been clearly established.

Most observational studies have shown that patients with specific dermatitis often have intestinal flora disturbance, but this may only be a clinical symptom of specific dermatitis and cannot prove a causal relationship (observational studies may have differences in intestinal flora due to grouping requirements, sex ratio, ethnicity, etc.). Therefore, MR is adopted to eliminate the interference of confounding factors on the one hand and avoid the influence of reverse causality on the other hand, so as to make the study more rigorous and credible (22).

From a genetic perspective, Mendelian randomization (MR) utilizes the genetic law of random distribution of gamete alleles to analyze potential causal relationships between exposures and outcomes. MR requires a genetic variant that is robustly associated with exposure as an instrumental variable, and the genetic variants have and can only act on the outcome indicators by influencing the exposure factors, largely reducing the interference of confounding and reverse causal associations. In this study, the two-sample Mendelian randomization was used to analyze the potential causal relationship between the gut microbiota and AD, using the abundance of the gut microbiota as the exposure factor and the occurrence of AD as the outcome, and explore the relationship between them genetically.

## 2. Materials and methods

### 2.1. Study design

**Figure 1:**
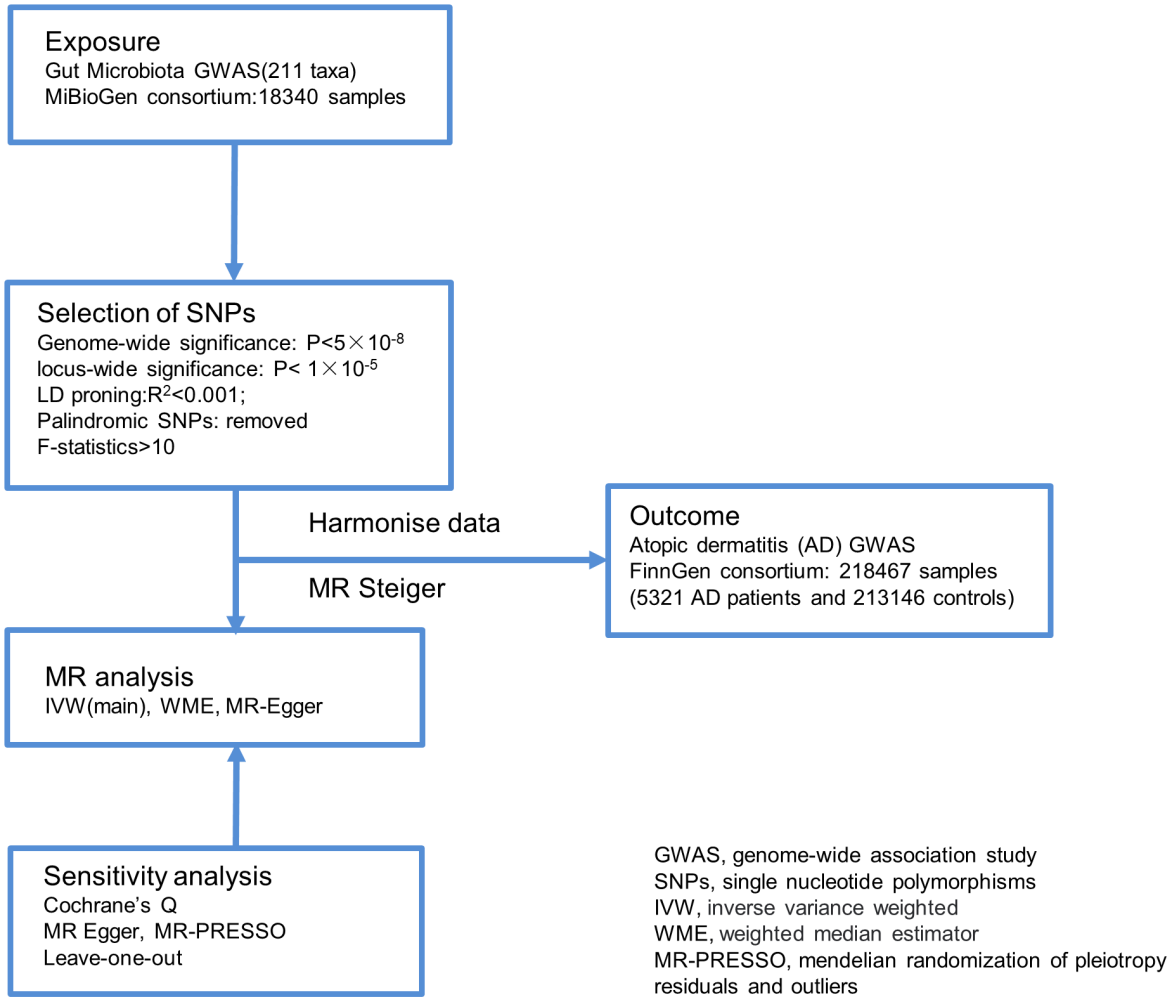
Overview of the analysis process of the causal relationship between the gut microbiome and Atopic dermatitis through MR analyzes. GWAS, genome-wide association study; SNPs, single nucleotide polymorphisms; IVW, inverse variance weighted; WME, weighted median estimator; MR-PRESSO, mendelian randomization of pleiotropy residuals and outliers.

This study utilized gut microbiota abundance as an exposure factor, and single nucleotide polymorphisms (SNPs) screened separately under the comprehensive threshold (1 × 10^−5^) and the traditional threshold (5 × 10^−8^) were used as instrumental variables (IVs).The outcome measure was atopic dermatitis (AD). Causal analysis was performed using a two-sample MR analysis approach (Figure 1).

### 2.2. Data sources

Both gut microbiota and AD data were obtained from genome-wide association study (GWAS) datasets. The intestinal microbiome data were derived from a large MiBioGen consortium GWAS analysis of 18,340 people, including 24 cohorts for whole-genome genotypes and 16S fecal microbiome data; the AD data were derived from strictly defined AD data collected by FinnGen biobank analysis, consisting of 5,321 AD patients and 213,146 controls (European descent).

### 2.3. Outcome measure

The current diagnostic criteria for AD are not uniform (23–25). This case refers to the Hanifin and Rajka diagnostic criteria (gold standard).

Hanifin and Rajka’s diagnostic criteria include four cardinal and 23 minor features (26). The basic characteristics are: 1. Itching; 2. Typical shape and distribution of the rash: children with facial and extensors involved; 3. Chronic or chronic recurrent dermatitis; 4. Personal or family history of atopic disease. The diagnosis of atopic dermatitis in children requires meeting any three of the basic characteristics plus any three of the secondary characteristics (Supplementary Table S1).

### 2.4. Instrumental variables

First, for the 1,000 Genomes project, we were originally going to choose SNPs that were significantly associated with gut microbiota (*p* < 5 × 10^−8^) of SNPs. If the number of screening is relatively small under the traditional threshold, a relaxation statistical threshold(*p* < 1 × 10^−5^) was commonly implemented in MR studies (27–30) to account for greater variation when few genome-wide significant SNPs were available for exposure (30–32). We set the threshold of *R*^2^ as 0.001, KB = 10,000 (clump step was performed using two-sample MR of R software to remove SNPs within 10 MB that were in LD with the most significant SNP with an R^2^ of more than 0.001 to exclude linkage disequilibrium effects). Second, based on the principle that the effects of the selected SNPs on exposure and outcome are due to the same alleles, palindromic SNPs that do not possess the A/T or C/G polymorphisms were excluded from IVs (33). The proportion of variation (*R*^2^) explained by SNPs in the exposure database was calculated by the following formula: *R*^2^ = 2 × β^2^ × (1-EAF) × EAF. In this formula, β identifies the estimated effect of the genetic variant and EAF represents the effect allele frequency, and then F = *R*^2^ (N-k-1)/k(1-R^2^) was calculated based on the sample size (N), the number of included SNPs (k), and *R*^2^ (34).When the F-statistic >10, then indicating the absence of instrumental variable bias [F for a single SNP equals β ^2^/SE^2^ (35)].

### 2.5. Mendelian randomization analysis

Inverse variance weighted (IVW), MR-Egger regression (36), weighted median estimator (WME) and Wald ratio was used for MR Analysis. The IVW method suggests that there is no level of pleiotropy, which avoids the influence of confounding factors to a certain extent and thus gets unbiased estimation (27, 37). The applicability of the MR-Egger hypothesis is strong, and it can withstand the polytropy of more than 50% SNP. However, WME was still applicable when the pleiotropy was less than 50%, which improved the accuracy of the results to some extent. In this study, IVW method was adopted as the primary causal effect estimation. IVW method is a relatively ideal estimation, which is an effective analysis under the basic premise that all genetic variations are valid instrumental variables, and has a strong ability to detect causality. Besides, another four methods were adopted to supplementary the results, namely MR-Egger regression, WME, simple modal-based estimation, and weighted modal-based estimation. It has been reported that, under certain conditions, the IVW method is more powerful and reliable than other methods (38). The Wald ratio (WR) method is often used when examining the effect of individual IV on causal estimation.

### 2.6. Horizontal pleiotropy and heterogeneity evaluation

Instrumental variables pleiotropy detection: the intercept term of MR-Egger regression tests for the presence of directional pleiotropy, if the intercept term egger intercept is close to zero, there is no pleiotropy for instrumental variables and vice versa. In addition, outliers can be detected for pleiotropy bias through Mendelian randomization of pleiotropy residuals and outliers (MR PRESSO) or by Cochran’s Q test to quantify the heterogeneity among the selected SNPs (*p* < 0.05 was considered as possible heterogeneity in IVs) (39). A leave-one-out sensitivity analysis was performed on the results by observing whether there was a statistical difference before and after removing each SNP one by one. If there is little change in the results before and after removing this SNP, which indicates that removing this SNP would maynot have a nonspecific effect on the effect estimate. MR Steiger test was used to determine the directivity of the impact of exposure on the outcome, and the result of “TRUE” predicts the association in the expected direction.The statistical treatments described above were carried out using R version 4.2.1 and implemented in the TwosampleMR package (version 0.5.6) with a 0.05 check level. Full documentation is available here: https://mrcieu.github.io/TwoSampleMR.

## 3. Results

### 3.1. Instrumental variables selection

**Figure 2:**
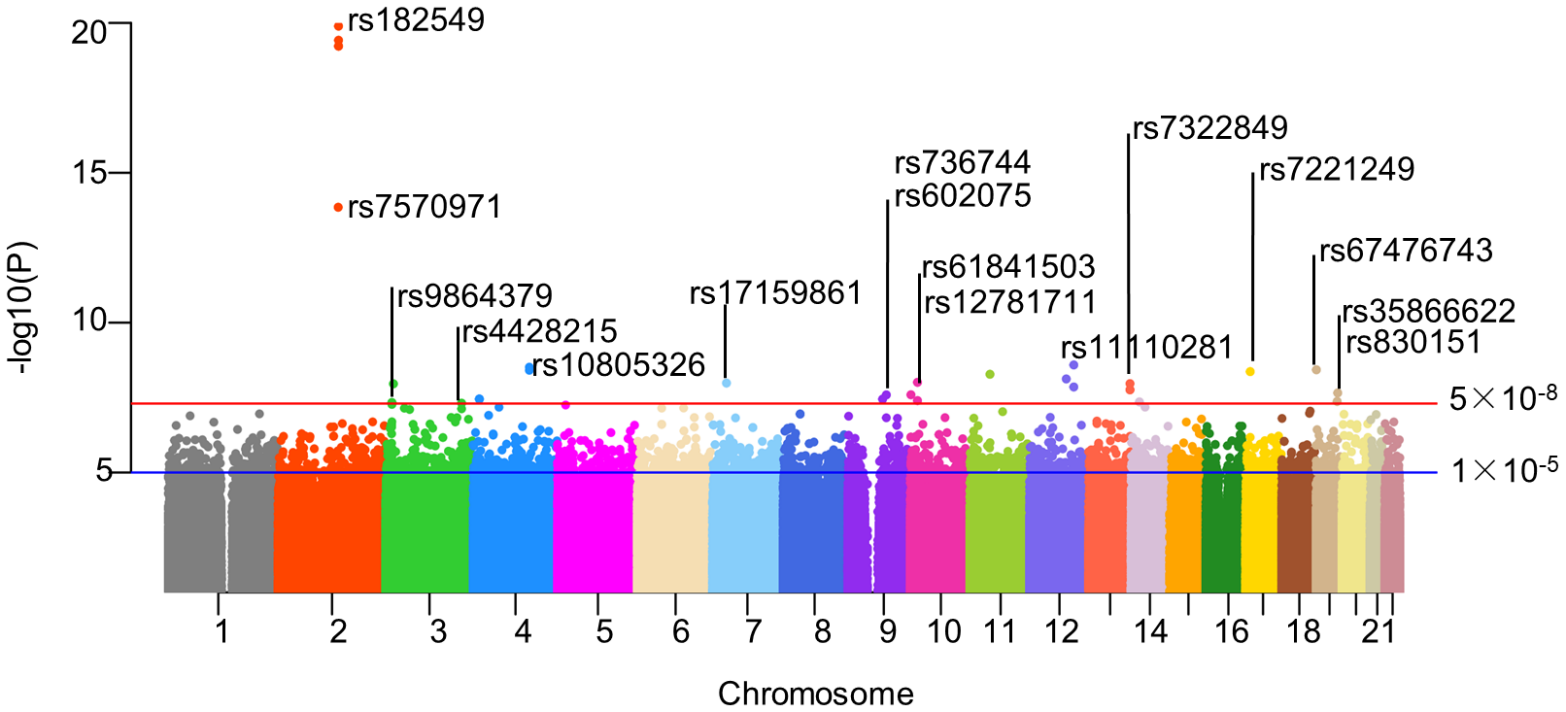
Manhattan plot of SNPs screening results. Horizontal lines define nominal genome-wide significance (*p* = 5 × 10^−8^. red) and suggestive genome-wide (*p* = 1 × 10^−5^. blue) thresholds. As described in our paper, 25 significant SNPs were included in our follow-up study after excluding SNP loci with palindromic structures under genome-wide association thresholds. Actinobacteria, Bifidobacteriaceae, *Bifidobacterium*, and Bifidobacteriales all contained rs182549, so we only identified them at the top of the corresponding SNPs. Similarly, rs61841503 (2), rs11110281 (2) and rs7322849 (3) were also identified only at the top. And the corresponding SNPs information could be read in the corresponding Supplementary Tables S2, S3. rs182549, rs7570971 (red); rs9864379, rs4428215 (limegreen); rs10805326 (dodgerblue); rs17159861 (lightskyblue); rs736744, rs602075 (purple2); rs61841503, rs12781711 (maroon2); rs11110281 (slateblue2); rs7322849 (tomato1); rs7221249 (gold1); rs67476743, rs35866622, rs830151(tan).

The intestinal flora (exposure) and AD (outcome) used in this analysis were summarized data collected by the MiBioGen Consortium and the Fingen Biobank analysis, respectively. Gut microbiota exposure was obtained from 24 cohort studies in the United States, Canada, Israel, South Korea, Germany, Denmark, the Netherlands, Belgium, Sweden, Finland, and the United Kingdom. These data included 16 s ribosomal RNA gene sequencing profiles and genotyping data from 18,340 participants. Seven different fecal DNA extraction methods and three different 16S rRNA regions (V4 (13 cohorts), V3-V4 (6 cohorts), and V1-V2 (5 cohorts)) were used. The exposure data included 211 intestinal biological groups including *Bifidobacterium*, Bacteroides, Clostridia, etc., which broadly summarized the distribution of human intestinal flora and was used for subsequent analysis by many studies. Detailed information was obtained from the study of the scholar Kurilshikov (40). The outcome included 5,321 AD patients and 213,146 control members (European), with a total number of SNPS of 16,380,466.After removing IVs in linkage disequilibrium, a total of 2,289 SNPs (*p* < 1 × 10^−5^) were included, including 5 taxa and 17 bacterial characteristics (1 phylum, 3 classes, 1 order, 4 families, and 8 genera). In addition, we also collected more information about SNPs (such as effect alleles, beta, SE, and *p* values), and all F-statastics >10, as shown in the Supplementary Table S2. Under the traditional threshold value (*p* < 5 × 10^−8^), we screened only 25 SNPs through a series of quality control, including 5 taxa and 22 bacterial characteristics(1 phylum, 1 class, 2 orders, 5 families, and 13 genera), and all F statistics were greater than 10 (Figure 2; Supplementary Table S3).

### 3.2. Mendelian randomization analysis of gut microbiota and AD

#### 3.2.1. Mendelian randomization analysis of gut microbiota and AD obtained at suggestive genome-wide significance (1 × 10^−5^)

Based on several methods of MR analysis, we observed evidence of a potential causal association between gut microbiota and AD risk. Bounded by a significance of 0.05. The IVW analysis showed that Tenericutes, Mollicutes, Clostridia, Bifidobacteriaceae, Bifidobacteriales, *Bifidobacterium*, and Christensenellaceae R 7 group were negatively correlated with the risk of AD (Figure 3), while Clostridiaceae_1, Bacteroidaceae, Bacteroides, Anaerotruncus, unknown genus, and Lachnospiraceae UCG001 showed the opposite trend (Figure 4; Supplementary Figures S1–S13).

**Figure 3:**
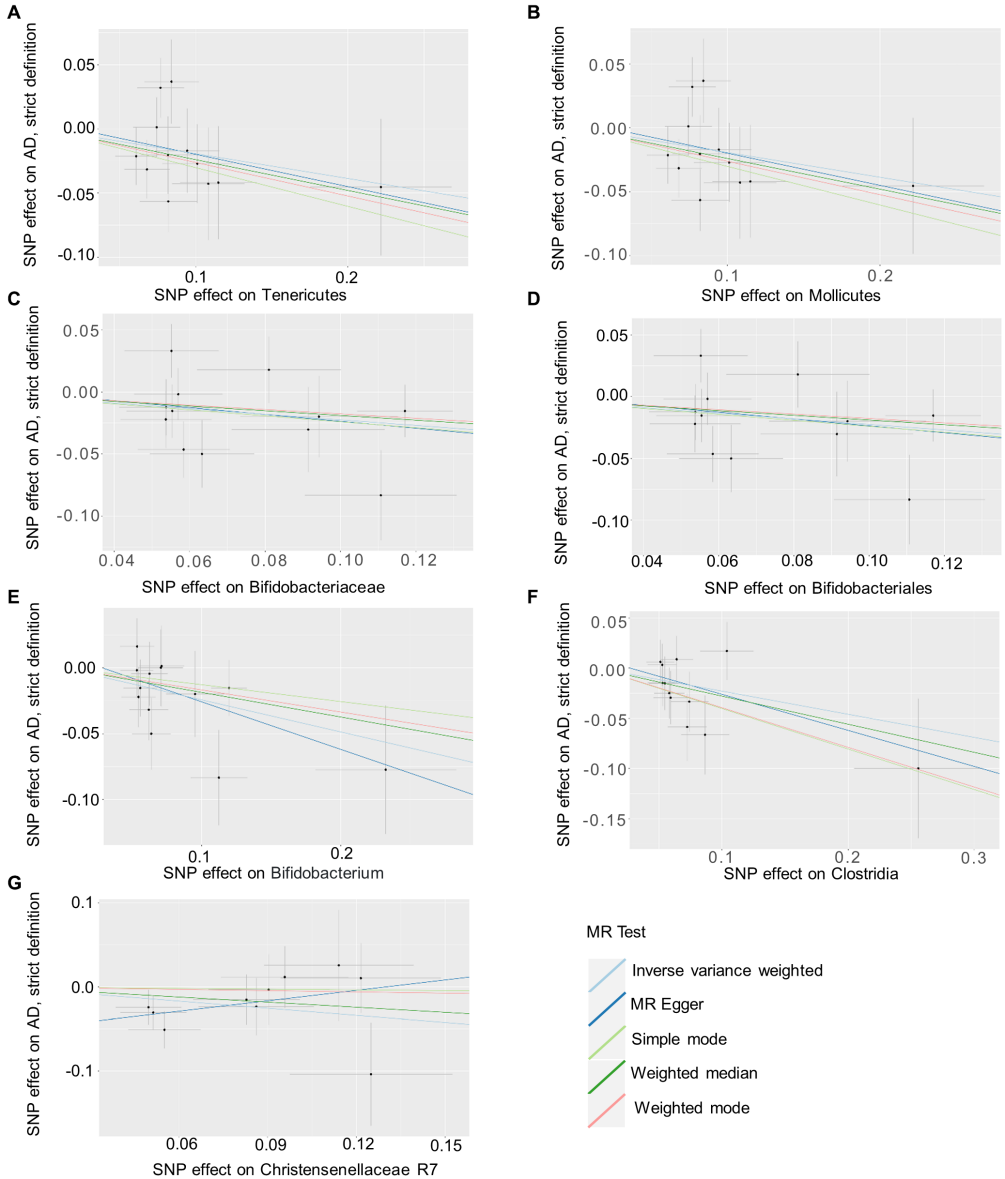
Summary of scatter plots of potential negative associations between intestinal flora and AD risk **(A–G)**. Each dot in the graph represents an SNP locus. The vertical axis of the graph is the effect of the instrumental variable on the outcome, the horizontal axis is the effect of the instrumental variable on the exposure, and the ratio of the two effects is the effect of exposure on the outcome, that is, the slope of the regression line corresponds to the causal effect of exposure on the outcome in the graph. Horizontal and vertical crosses show a 95% confidence interval for each association. The estimates for the MR Analysis were slightly different, but the overall downward-sloping trend suggests that exposure (this gut microbiome) may have a negative causal effect on the outcome (AD). AD, atopic dermatitis; SNPs, single nucleotide polymorphisms; MR, Mendelian randomization; IVW, inverse variance weighted.

**Figure 4:**
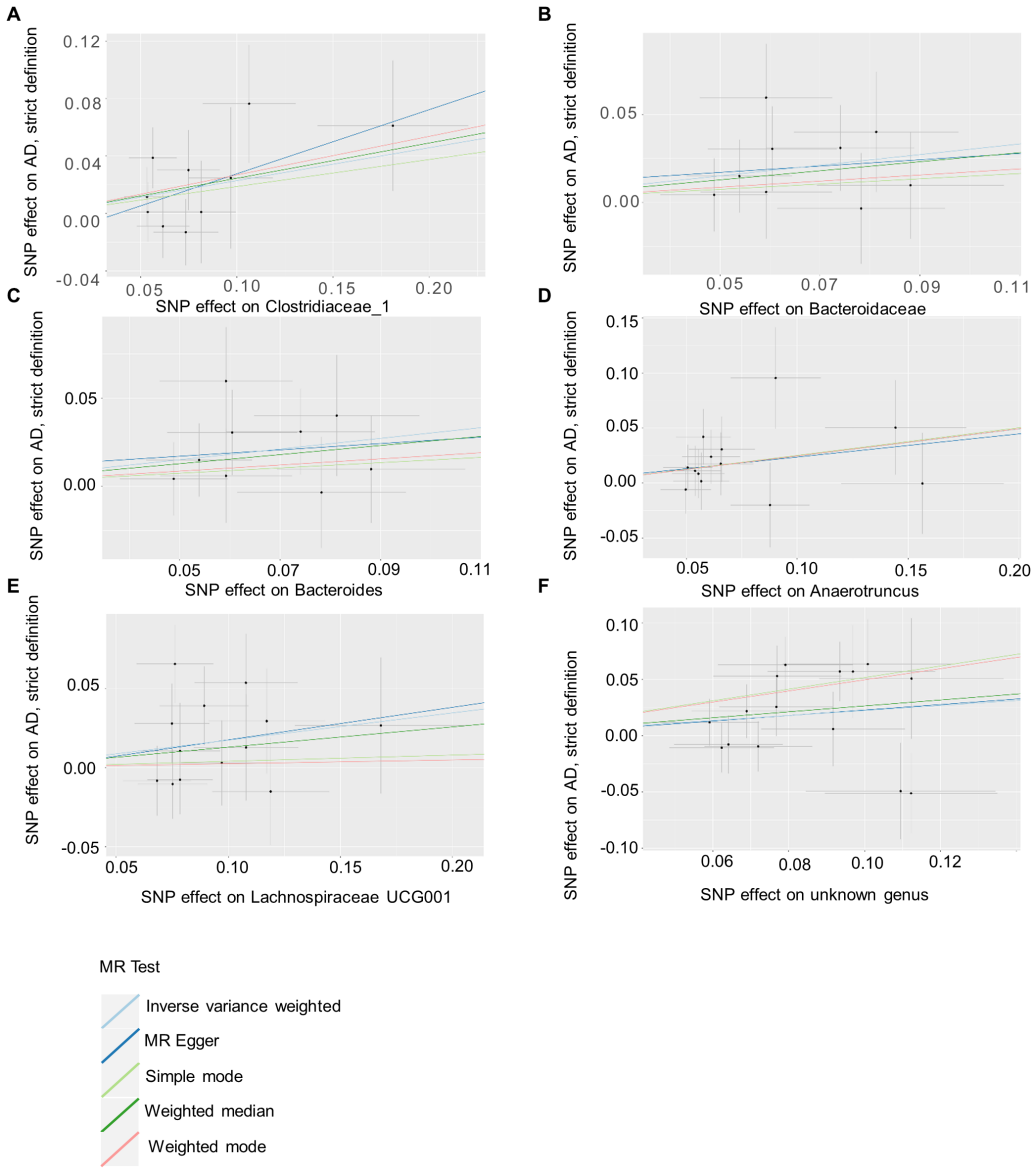
Summary of scatter plots of potential positive associations between intestinal flora and AD risk **(A–F)**. IVW estimates show that Clostridiaceae_1, Bacteroidaceae, Bacteroides, Anaerotruncus, unknown genus, and Lachnospiraceae UCG001 and AD show an upward trend, suggesting a potential positive correlation between them. AD, atopic dermatitis; SNPs, single nucleotide polymorphisms; MR, Mendelian randomization; IVW, inverse variance weighted.

**Figure 5:**
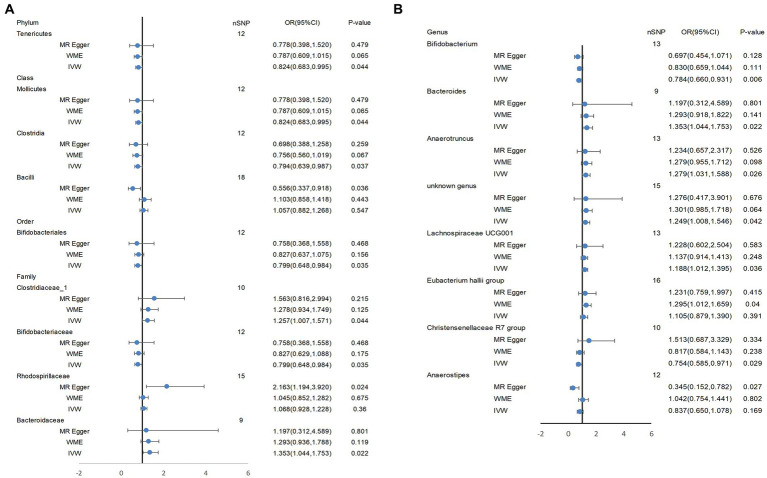
Tenericutes, Mollicutes, Clostridia, Bifidobacteriaceae, Bifidobacteriales, *Bifidobacterium*, and Christensenellaceae R 7 group, Clostridiaceae_1, Bacteroidaceae, Bacteroides, Anaerotruncus, unknown genus, and Lachnospiraceae UCG001 showed a potential causal relationship with AD risk. In **(A)**, the three most classical MR Analysis methods are used to demonstrate the potential causal relationship between some bacterial groups and AD risk at four levels (phylum, class, order, family). **(B)** shows the potential causal association at the genus level between them. IVW, inverse variance weighted; WME, weighted median estimator.

In addition, WME analysis results suggested that the *Eubacterium hallii* group (OR = 1.295, 95%CI, 1.012–1.659, *p* = 0.040; Supplementary Figure S14) was a risk factor for AD. MR-Egger estimated that genetically related Rhodospirillaceae were positively correlated with AD risk (OR = 2.163, 95%CI, 1.194–3.920, *p* = 0.024; Supplementary Figure S15), and Bacilli (OR = 0.556, 95%CI, 0.337–0.918, *p* = 0.036, Supplementary Figure S16), Anaerostipes(OR = 0.345, 95%CI, 0.152–0.782, *p* = 0.027; Supplementary Figure S17) had a potential protective effect on AD (Figure 5; Supplementary Table S4). The detailed information on the included SNPs were shown in the Supplementary Table S2.

MR-Egger regression estimated that the *p* values of the horizontal pleiotropy results for the three kinds of bacteria were all less than 0.05, but the MR-PRESSO test results suggested that there was no horizontal pleiotropy and outlier values, which need further discussion here. In addition, no horizontal pleiotropy was found in the other bacteria. The statistical values of SNPs were all greater than 10, indicating that there was no weak IVs bias in this study, and the analysis results were more credible. In addition, no SNPs that had a significant effect on causal association were found in the leave-one-out analysis (Supplementary Table S4). After MR Analysis of intestinal flora and atopic dermatitis, we concluded that significant matches between 2 **×** 10^−4^ (0.05/211) and 0.05 of intestinal flora may have a potential causal association with atopic dermatitis to some extent (29). MR Steiger’s test verified the causal hypothesis between intestinal flora exposure and AD, and the results showed that the effect of intestinal flora on AD was in a expected causal direction.

#### 3.2.2. Mendelian randomization analysis of gut microbiota and AD obtained at nominal genome-wide significance (5 × 10^−8^)

The MR Results included a total of 22 gut microbiota abundance information related to genetic prediction, including 1 phylum (1 SNP), 1 class (1 SNP), 2 orders (3 SNPs), 5 families (6 SNPs), and 13 genera (14 SNPs), whose details were shown in Supplementary Table S3. Unknown genus, unknown family, and order Gastranaerophilales (all rs9864379) might be positively associated with AD risk (Wald ratio: OR,95%CI = 1.509 (1.057, 2.152), *p* = 0.023), as shown in Supplementary Table S5. The remaining taxa did not show a potential causal relationship with AD risk. Moreover, due to the small number of SNPs included under each gut microbiota category (1 or 2 SNPs), it was not possible to perform subsequent sensitivity analysis.

## 4. Discussion

Normal gut microbiology is mostly composed of firmicutes, bacteroidetes, actinobacteria, and proteobacteria (29), which remain relatively conserved and stable at the phylum level but differ considerably at the species level. We found in this MR analysis that Tenericutes, Mollicutes, Clostridia, Bifidobacteriaceae, Bifidobacteriales, *Bifidobacterium*, Christensenellaceae R7, Bacilli, and Anaerostipes levels were inversely associated with the risk of AD, and *Eubacterium hallii* group, Clostridiaceae_1, Bacteroidaceae, Bacteroides, Anaerotruncus, unknown genus, and Lachnospiraceae UCG 001 were potential risk factors for AD.

The gut microbiota plays an important role not only in immune, and metabolic regulation but also in neurodevelopment and feedback mechanisms, among others, from the period when the infant begins colonization after birth until the entire human life span (41). Some studies have found that gut microbiota diversity and abundance of specific bacteria are closely related to the onset age and severity of AD, but some scholars also do not recognize the former view. Therefore, whether gut microbiota abundance change is a key reason for the occurrence and development of AD becomes the main entry point for this MR.

Colonization with specific flora may interfere with the course of AD by affecting the intestinal barrier and the balance of intestinal microbiology. For example, a birth cohort for childhood origin of asthma and allergic diseases (COCOA) found that low levels of Streptococcus and high levels of Akkermansia (Akkermansia family, Verrucomicrobia order) were found in transient AD cases, while the opposite was true in AD children with a persistent system (42). Furthermore, the abundance of the Clostridium genus increased, while the abundance of gut microbial functional genes related to energy metabolism and SCFA decreased. Lee reached a similar conclusion through metagenomic analysis (9).

Surprisingly, certain specific gut flora associated with AD also play important roles in cognitively dysfunctional populations, further driving the hypothesis of a “gut-brain-skin axis” (43). Increased abundances of Clostridiaceae were also strongly associated with AD risk in our analysis (Supplementary Table S4), among which increased abundance of clostridia is strongly associated with AD risk, possibly through the release of toxins that inhibit the chemotaxis of neutrophils and inactivate eosinophils, aggravating intestinal inflammation. On the one hand, the impaired intestinal barrier enters the bloodstream by releasing substances such as tryptophan and serotonin, and atopic march to distant skin (44). On the other hand, activates neuroimmune pathways to transmit signals to the brain, causing emotions such as anxiety and depression, and further releases neurotransmitters such as 5-hydroxytryptamine (5-HT, also known as serotonin) and tryptophan to affect the status of distant skin. The findings support the hypothesis of the “gut-skin-axis” and “gut-brain-skin axis” and add to the potential mechanisms explaining the causal association of gut flora with AD risk (Supplementary Figure S18). We found that, Anaerostipes could be used as a potential protective factor for AD in this study. Similar to what was reported by scholar Taylor, Anaerostipes could participate in the regulation of food anti-allergic reaction (45), and protect the body from the attack of foreign antigens. Rhodospirllaceae belongs to the alpha-proteus family, and it has been found that Rhodospirllaceae is involved in the pathogenesis of cognitive function and neuropsychiatric symptoms of Alzheimer’s disease (46), suggesting the potential background of Rhodospirllaceae in the gut-brain axis. In this paper, it was also found that Rhodospirllaceae is closely related to the pathogenesis of AD. It provides a new way of thinking for us to search for the specific mechanism of the “gut-brain-skin axis.”

In addition, it has also been reported that Enterococcus and Shigella are also significantly higher in children with early AD and that serotonin production aggravates skin pigmentation, whereas *Bifidobacterium* abundance decreases (47). As well-known probiotics, Bifidobacteria and *Bifidobacterium* play important roles in protein synthesis, preventing the invasion of foreign bacteria, and stimulating immunity (48). Bifidobacteria, on the other hand, can promote Treg differentiation while inhibiting the Th2 response. It also increases the proportion of *Lactobacillus* positively associated with increased propionic acid production (49, 50), on the other hand, it alleviates AD symptoms by regulating tryptophan metabolites (indole derivatives) to activate the aryl hydrocarbon receptor (AHR) signaling pathway (44). Bacilli include Bacillus and *Lactobacillus*, of which *Lactobacillus* has been reported to involved in eosinophilic regulation, reducing eosinophil count and serum IgE concentration, activating regulatory T cells and TH1/TH2 balance, and contributing to intestinal microecological balance (51–53). Like *Lactobacillus*, *Bacillus* subgroups also play a protective role by regulating intestinal microecology. Some of the Bacillus species (such as *Bacillus coagulans* TL3, *Bacillus velezensis* A2) can reduce oxidative stress and inflammatory damage by participating in the regulation of signaling pathways, and also participate in the regulation of intestinal flora abundance, increasing the relative abundance of beneficial bacteria and the expression of intestinal barrier tight junction protein (54–56).

Christensenellaceae R7 belongs to the phylum firmicutes and is widely found in the intestinal mucosa of humans and animals. Some scholars have found that Christensenellaceae R7 is significantly negatively correlated with Body Mass Index (BMI) (57), inflammation (58), and other metabolic diseases (59, 60). Similarly, when scholar Zhao (61) established acute colitis models in sensitized pigs and Yorkshire pigs, he found potentially beneficial microorganisms related to intestinal barrier function, such as *Lactobacillus*, Eubacillus, and Christensenellaceae, through microbial sequencing analysis. Once again, the potential protective effect of Christensenellaceae was verified. Desulfurizing bacteria, Bacteroides, and *Streptococcus* still play dangerous roles, as discussed earlier. In the results of this study, the *Eubacterium hallii* group is considered a risk factor for AD, which has been proven in many autoimmune diseases such as Hashimoto’s thyroiditis (62), clear cell renal cell carcinoma (63), rheumatoid arthritis (RA) (64). *Eubacterium hallii* group and Lachnospiraceae UCG001 belong to the Lachnospiraceae family and can both produce butyrate and participate in the metabolism of amino acids and lipids. In the study of intestinal flora dysbiosis and hyperuricemia, scholar Song found that the enrichment of Lachnospiraceae UCG001 and Anaerotruncus led to intestinal dysbiosisand intestinal barrier damage, which interfered with amino acid metabolism and increased blood uric acid level and CD4 Th17-driven inflammation (65).

In addition, we found that as the risk of AD increased, the abundance of Bacteroidaceae and Bacteroides gradually increased. In line with our findings, the author Siqi Ye mentioned that the α diversity of intestinal flora in AD patients was decreased compared with healthy controls, but the relative abundance of parabacteroides, Bacteroides ovoides, and homobacteroides was significantly increased (66) The change in Bacteroides abundance is closely associated with gout (67), inflammatory bowel disease (68), autoimmune thyroid disease (AITD) (69), multiple sclerosis (MS) (66), rheumatoid arthritis (RA) (70), etc. Therefore, we speculate that Bacteroides may be used as diagnostic markers for autoimmune diseases such as AD in the future.

Tenericutes belong to the one of the placental-specific flora,part of which is involved in the composition of non-pathogenic symbiotic microbiota (71). Tenericutes may show different associations with health and disease (72–74). Although it is considered in this study that Tenericutes and Mollicutes mayhave potential protective effects on AD, the correlation effect of pairing them has not been observed at a more detailed level, so deeper studies are needed to explain clearly.

The microbiome may be a target for the treatment of immune diseases (75). Most observational studies believe that SCFA (such as acetate, butyrate, and kynurenic acid) produced by Akkermansia, Bifidobacteria, Facalibacterium, and other bacteria in the gut. is closely related to the diversity of the skin microbiome (76–78). SCFAs inhibit immune response by inhibiting the proliferation of inflammatory cells and the production of cytokines. In addition, SCFAs also regulate apoptosis and activation of immune cells by inhibiting histone deacetylase (HDAC) (79).

Therefore, it is intended to correlate the changes in intestinal microbiome abundance with AD, psoriasis, common eczema, and other skin lesions and develop new immunomodulators to promote skin improvement. In our analysis, it can also be observed that Bacteroides, Lachnospiraceae, and Bifidobacteriaceae(these three intestinal bacteria are related to SCFA synthesis) have a potential causal relationship with AD. Song et al. combined with whole genome sequencing did not observe the reduction of evolutionary branches of *Lactobacillus* and *Bifidobacterium* in the analysis (80), and some reports believed that the increase of some groups in the intestinal flora of children with AD was not related to AD, but to the potential harmful flora in the damaged intestinal environment (81). For acute AD, changes in the composition of some intestinal flora may be less obvious, leading to different conclusions. In order to avoid the interference of confounding factors, we strictly abide by the three principles of MR and verify the causal association between the changes in the abundance of intestinal flora and the risk of AD from the genetic perspective.

Some researchers found that Gastranaerophilales was one of the main indole-producing bacteria, and the concentration of indole and its derivatives was increased in patients with Alzheimer’s disease (82), suggesting that this bacterium might be involved in the formation of the gut-brain axis. Furthermore, scholar wang used enterotoxigenic *Escherichia coli* (ETEC) to damage the intestinal epithelial barrier function, causing severe intestinal diarrhea in mice. In the feces of the disease group, the abundance of pathogenic bacteria(Gastranaerophilales and *Escherichia coli*) in the intestine was found to increase (83). In a study based on a GWAS dataset, Cao and colleagues used microbiota-related gene set enrichment analysis to explore the relationship between the gut microbiota and six autoimmune diseases, including chylous diarrhea (CeD), inflammatory bowel disease (IBD), multiple sclerosis (MS), primary biliary cirrhosis (PBC), type 1 diabetes (T1D), and primary sclerosing cholangitis (PSC), and osteoporosis (OP). Surprisingly, Peptostreptococcaceae, Gastanaerophilales, and Romboutsia were found in the flora of the other six autoimmune diseases except for PBC (84). In addition, there was a possible association between AD and autoimmune diseases (85), so we suspected that Gastranaerophilales may also be involved in the pathogenesis of AD.

Host genetics may also play an important role in the construction of intestinal flora. Host genetic distance was found to be associated with BMI and the combination of intestinal flora diversity (86), and animal experiments also identified genes involved in metabolism, immunity, and behavior, such as myeloid differentiation primary response protein 88 (MYD88) (87), human leukocyte antigen (HLA) may be associated with intestinal flora (88). The combination of environment and heredity on intestinal flora provides an idea for further understanding of “gut-skin” axis.

To the best of our knowledge, this is the first MR study to look at the genetic link between the gut microbiome and atopic dermatitis. According to this MR analysis, we recognized the causal relationship between intestinal flora and AD and found that some specific intestinal flora participated in the pathogenesis of AD. The present MR analysis of the causal relationship between the gut microbiota and AD, which both reduces the possibility of the presence of confounders in the outcome and negates the reverse causal association of the two, is more convincing than observational studies.

But in fact, there are still some deficiencies: First, the data included in this MR analysis are all of European ancestry, and different ethnicities do not share the same dominant gut flora due to different exposure factors such as dietary environment (9), so generalizing the results to other ethnic populations is limited; Second, we adopted linear MR analysis because specific sample data were not available, resulting in the inability to perform subgroup analyzes based on age and gender; Then, we included SNPs of gut microbiota at the phylum, class, order, family, and genus levels, which could not be analyzed at a more specific species level. What’s more, because our study was based on GWAS summarized data, we were unable to obtain detailed clinical differences within each group of patients with specific dermatitis, and therefore could not combine clinical differences among each group for further study. Finally, the composition of the infant gut microbiota is highly dynamic, with certain species genera exhibiting opposite states at the beginning versus the continuing stages of AD, but our original hypothesis is that the emergence and change of certain species abundances is relatively stable.

## 5. Conclusion

Using a two-sample Mendelian randomization approach, our study provides a potential causal association between the abundance of gut microbes and the risk of AD and suggests a genetic relationship between the two. The results of our MR analysis will provide beneficial support for gut microecological-based therapy of AD and lay a solid foundation for further exploration of the pathogenesis of gut microbiota leading to AD.

## Data availability statement

The datasets presented in this study can be found in online repositories. The names of the repository/repositories and accession number(s) can be found in the article/Supplementary material.

## Author contributions

YX, LZ, YC, HW, and JX: study design. YX, HW, LZ, and YC: data collection and data analysis. YX, LZ, and YC: drafting manuscript. All authors take responsibility for the integrity of the data analysis and read and approved the article.

## Funding

This work was funded by the Science and Technology Planning Project of Sichuan Province (2021YJ0170) and the Project of Chengdu Science and Technology Bureau (2019-YF05-00498-SN).

## Conflict of interest

The authors declare that the research was conducted in the absence of any commercial or financial relationships that could be construed as a potential conflict of interest.

## Publisher’s note

All claims expressed in this article are solely those of the authors and do not necessarily represent those of their affiliated organizations, or those of the publisher, the editors and the reviewers. Any product that may be evaluated in this article, or claim that may be made by its manufacturer, is not guaranteed or endorsed by the publisher.
