# Supplementary material for: Gut microbiota and atopic dermatitis: a two-sample Mendelian randomization study
Source: Front Med (Lausanne). 2023 Jun 22;10:1174331. doi: 10.3389/fmed.2023.1174331 (PMC10323683; doi:10.3389/fmed.2023.1174331)
Supplement: Supplementary file 1 [file Table_1.DOCX]

- **Supplementary Table 1. Diagnostic criterias of Hanifin and Rajka.**

| Main criterias(4) | Itching  Typical shape and distribution of the rash  Chronic or chronic recurrent dermatitis  Personal or family history of atopic disease |
| --- | --- |
| Minor criterias(23) | Asteatosis cutis |
|  | Crothyosis/Palmotopy/Perihairy keratosis |
|  | Type I skin test reaction |
|  | Elevated serum IgE |
|  | Early onset age |
|  | Skin infection propensity/impaired cell-mediated immunity |
|  | Tendency toward cutaneous infections |
|  | Eczemaofnipple |
|  | Cheilitis |
|  | Recurrent conjunctivitis |
|  | Denny Morgan infraorbital fold |
|  | Keratoconus |
|  | Anterior subcapsular cataract |
|  | Peri-orbit halo |
|  | Pale face/facial erythema |
|  | Pityriasis alba |
|  | Perifollicular accentuation |
|  | Itching when you sweat |
|  | Intolerance to solvents such as wool |
|  | Beely uplift |
|  | Food intolerance |
|  | The course of disease is influenced by the environment |
|  | White scratch sign/delayed whitening |
