# Supplementary material for: Gut microbiota and atopic dermatitis: a two-sample Mendelian randomization study
Source: Front Med (Lausanne). 2023 Jun 22;10:1174331. doi: 10.3389/fmed.2023.1174331 (PMC10323683; doi:10.3389/fmed.2023.1174331)
Supplement: Supplementary file 2 [file Table_2.DOCX]

Supplementary Table 2. SNPs were used as instrumental variables from gut microbiome and AD GWASs (P <1 ×10^-5^).

| Name | N | SNP | CHR | Position | beta. exposure | se. exposure | beta. outcome | se. outcome | F |
| --- | --- | --- | --- | --- | --- | --- | --- | --- | --- |
| class Bacilli (ebi-a-GCST6910) | 1 | rs11110282 | 12 | 100585559 | -0.10119 | 0.021738 | -0.0013 | 0.0486 | 21.66863 |
|  | 2 | rs11730038 | 4 | 98049499 | -0.06307 | 0.012872 | -0.0152 | 0.0227 | 24.01257 |
|  | 3 | rs12797734 | 11 | 8332350 | 0.057216 | 0.012679 | 0.0446 | 0.0239 | 20.36298 |
|  | 4 | rs13068444 | 3 | 64398863 | 0.060104 | 0.013641 | -0.0054 | 0.0271 | 19.4145 |
|  | 5 | rs1595463 | 2 | 231723657 | 0.047686 | 0.010855 | 0.0184 | 0.0209 | 19.29983 |
|  | 6 | rs28564647 | 9 | 101101172 | -0.0613 | 0.013758 | 0.0199 | 0.0277 | 19.85477 |
|  | 7 | rs2952251 | 8 | 10143164 | 0.059887 | 0.01239 | 0.0031 | 0.027 | 23.36138 |
|  | 8 | rs34989881 | 19 | 51959855 | 0.111056 | 0.024604 | 0.0217 | 0.05 | 20.37399 |
|  | 9 | rs35344081 | 16 | 991253 | 0.061953 | 0.012707 | 0.0207 | 0.0235 | 23.77194 |
|  | 10 | rs4028634 | 17 | 40835649 | -0.05206 | 0.010984 | -0.0247 | 0.0214 | 22.4644 |
|  | 11 | rs4459992 | 4 | 7431487 | 0.053585 | 0.011636 | 0.0362 | 0.022 | 21.20658 |
|  | 12 | rs57872228 | 1 | 200418805 | -0.07148 | 0.014665 | -0.0079 | 0.0326 | 23.75691 |
|  | 13 | rs694949 | 15 | 58738075 | -0.08115 | 0.018004 | 0.0269 | 0.0352 | 20.31359 |
|  | 14 | rs74663707 | 3 | 184371224 | 0.097915 | 0.022407 | 0.0118 | 0.0421 | 19.09497 |
|  | 15 | rs7666190 | 4 | 151687420 | 0.104044 | 0.024793 | -0.0315 | 0.032 | 17.6101 |
|  | 16 | rs77558518 | 5 | 174173171 | -0.10726 | 0.022288 | -0.0114 | 0.0352 | 23.15777 |
|  | 17 | rs78938557 | 7 | 36349586 | 0.108007 | 0.023286 | -0.0571 | 0.062 | 21.51402 |
|  | 18 | rs9581006 | 13 | 24973509 | -0.22531 | 0.04677 | 0.0794 | 0.0554 | 23.20748 |
| class Clostridia (ebi-1-GCST90016913) | 1 | rs10774377 | 12 | 5942519 | -0.05266 | 0.011386 | -0.0031 | 0.0209 | 21.39036 |
|  | 2 | rs112334273 | 21 | 40703251 | 0.064101 | 0.01274 | 0.0087 | 0.0231 | 25.314 |
|  | 3 | rs13105690 | 4 | 7420184 | 0.05291 | 0.011818 | -0.0148 | 0.023 | 20.04392 |
|  | 4 | rs13179700 | 5 | 149077788 | -0.05119 | 0.010957 | -0.0061 | 0.0218 | 21.8302 |
|  | 5 | rs1842454 | 5 | 105060362 | -0.05472 | 0.012734 | 0.0152 | 0.0266 | 18.46628 |
|  | 6 | rs2273429 | 14 | 52494072 | -0.0724 | 0.015311 | 0.0587 | 0.0336 | 22.36365 |
|  | 7 | rs6797343 | 3 | 89268714 | -0.05923 | 0.013456 | 0.0295 | 0.0263 | 19.37522 |
|  | 8 | rs6814436 | 4 | 161507301 | -0.074 | 0.015082 | 0.0335 | 0.0297 | 24.07512 |
|  | 9 | rs6815608 | 4 | 152131744 | -0.1038 | 0.021088 | -0.017 | 0.0287 | 24.22907 |
|  | 10 | rs72738886 | 5 | 35770550 | 0.086617 | 0.019039 | -0.0665 | 0.0393 | 20.69681 |
|  | 11 | rs72915163 | 18 | 48792829 | -0.0581 | 0.012056 | 0.025 | 0.0253 | 23.22762 |
|  | 12 | rs992074 | 21 | 18567802 | -0.25564 | 0.050879 | 0.1 | 0.0695 | 25.2454 |
| class Mollicutes (ebi-a-GCST90016921) | 1 | rs10108398 | 8 | 59440824 | 0.076914 | 0.015395 | 0.032 | 0.0231 | 24.95955 |
|  | 2 | rs11890098 | 2 | 157532549 | 0.074438 | 0.015339 | 0.0012 | 0.023 | 23.55054 |
|  | 3 | rs12566890 | 1 | 61850864 | -0.10115 | 0.023098 | 0.0271 | 0.0308 | 19.1763 |
|  | 4 | rs17214486 | 14 | 97159674 | 0.060990 | 0.013562 | -0.0214 | 0.0222 | 20.22323 |
|  | 5 | rs2464826 | 7 | 79490250 | 0.094424 | 0.021181 | -0.017 | 0.0327 | 19.87409 |
|  | 6 | rs28537087 | 15 | 95309676 | 0.082087 | 0.018831 | -0.0565 | 0.024 | 19.00185 |
|  | 7 | rs3768491 | 1 | 109965986 | 0.068105 | 0.014906 | -0.0316 | 0.0227 | 20.87529 |
|  | 8 | rs4885016 | 13 | 73170489 | 0.081961 | 0.018163 | -0.0204 | 0.0301 | 20.36314 |
|  | 9 | rs6043847 | 20 | 16259524 | -0.11494 | 0.024861 | 0.0419 | 0.0439 | 21.37453 |
|  | 10 | rs72901605 | 11 | 47103877 | -0.08419 | 0.017812 | -0.0368 | 0.0327 | 22.33835 |
|  | 11 | rs74603314 | 14 | 46519718 | 0.221639 | 0.046292 | -0.0454 | 0.0531 | 22.92367 |
|  | 12 | rs78169027 | 11 | 108439087 | -0.10828 | 0.023729 | 0.0428 | 0.0438 | 20.82406 |
| family Bacteroidaceae(ebi-a-GCST90016927) | 1 | rs11585893 | 1 | 10644351 | -0.07407 | 0.014763 | -0.031 | 0.0242 | 25.17513 |
|  | 2 | rs13207588 | 6 | 41519430 | -0.05921 | 0.01312 | -0.006 | 0.0264 | 20.3649 |
|  | 3 | rs1340391 | 1 | 102960989 | -0.05920 | 0.013224 | -0.0596 | 0.0304 | 20.03998 |
|  | 4 | rs17619981 | 19 | 24342250 | 0.088098 | 0.0187 | 0.0098 | 0.0303 | 22.1941 |
|  | 5 | rs2023437 | 14 | 22045949 | -0.07823 | 0.016763 | 0.0034 | 0.0314 | 21.77962 |
|  | 6 | rs66474973 | 20 | 58605432 | 0.081252 | 0.016448 | 0.0401 | 0.0341 | 24.40292 |
|  | 7 | rs66710942 | 3 | 77215327 | -0.0488 | 0.010741 | -0.0043 | 0.0207 | 20.64437 |
|  | 8 | rs6795673 | 3 | 10593224 | 0.053857 | 0.010525 | 0.015 | 0.0206 | 26.18326 |
|  | 9 | rs9507307 | 13 | 24910476 | 0.060446 | 0.012913 | 0.0305 | 0.024 | 21.91233 |
| family Bifidobacteriaceae (ebi-a-GCST90016929) | 1 | rs10831953 | 11 | 13098051 | 0.053754 | 0.012375 | -0.0124 | 0.0225 | 18.86924 |
|  | 2 | rs12446429 | 16 | 898055 | 0.081010 | 0.019073 | 0.0179 | 0.0267 | 18.04007 |
|  | 3 | rs13020688 | 2 | 192878532 | 0.058405 | 0.012208 | -0.0464 | 0.0226 | 22.8874 |
|  | 4 | rs182549 | 2 | 136616754 | -0.11707 | 0.01267 | 0.0153 | 0.0210 | 85.37239 |
|  | 5 | rs4957061 | 5 | 521096 | 0.056987 | 0.011691 | -0.0018 | 0.021 | 23.76157 |
|  | 6 | rs540489 | 17 | 72897722 | -0.06325 | 0.013817 | 0.05 | 0.0271 | 20.95648 |
|  | 7 | rs55888705 | 4 | 1517826 | 0.053673 | 0.012059 | -0.0221 | 0.023 | 19.81153 |
|  | 8 | rs6899771 | 6 | 97406220 | -0.09143 | 0.02026 | 0.0303 | 0.0342 | 20.36539 |
|  | 9 | rs7174549 | 15 | 92463303 | -0.05517 | 0.012466 | -0.0331 | 0.0215 | 19.58969 |
|  | 10 | rs7322849 | 13 | 112859829 | 0.110676 | 0.0201 | -0.0833 | 0.0361 | 30.3202 |
|  | 11 | rs73797465 | 5 | 142793467 | -0.09426 | 0.020842 | 0.0199 | 0.0326 | 20.45422 |
|  | 12 | rs857444 | 6 | 14617591 | 0.055397 | 0.012067 | -0.0153 | 0.0214 | 21.07527 |
| family Clostridiaceae1 (ebi-a-GCST90016931) | 1 | rs10875374 | 1 | 101804809 | 0.053676 | 0.011944 | 0.0011 | 0.0207 | 20.19706 |
|  | 2 | rs12186080 | 3 | 132596627 | 0.074824 | 0.01624 | 0.0302 | 0.0278 | 21.21213 |
|  | 3 | rs12341505 | 9 | 136710881 | 0.08145 | 0.017934 | 0.0011 | 0.0355 | 20.62722 |
|  | 4 | rs2795528 | 10 | 43270264 | -0.18097 | 0.039091 | -0.0611 | 0.0452 | 21.4315 |
|  | 5 | rs2817172 | 1 | 3041519 | 0.056337 | 0.012392 | 0.0388 | 0.0212 | 20.66807 |
|  | 6 | rs4723021 | 7 | 30934659 | -0.10638 | 0.024194 | -0.0764 | 0.041 | 19.33115 |
|  | 7 | rs550843 | 6 | 165722832 | -0.07348 | 0.016838 | 0.013 | 0.023 | 19.04261 |
|  | 8 | rs56188186 | 16 | 87710690 | 0.096777 | 0.021747 | 0.0248 | 0.049 | 19.80361 |
|  | 9 | rs62397761 | 6 | 48093014 | 0.061581 | 0.01362 | -0.0089 | 0.0221 | 20.44302 |
|  | 10 | rs881532 | 22 | 47806774 | 0.053304 | 0.011901 | 0.0116 | 0.0206 | 20.06029 |
| family Rhodospirillaceae (ebi-a-GCST90016949) | 1 | rs1035406 | 5 | 119372737 | -0.11354 | 0.025086 | 0.0221 | 0.0323 | 20.48446 |
|  | 2 | rs11591293 | 10 | 113419797 | 0.074318 | 0.015872 | -0.0157 | 0.0209 | 21.92338 |
|  | 3 | rs13336560 | 16 | 88554243 | -0.07017 | 0.015806 | 0.0079 | 0.0211 | 19.70956 |
|  | 4 | rs1549633 | 5 | 27945645 | 0.099931 | 0.021863 | 0.0149 | 0.0323 | 20.89136 |
|  | 5 | rs1923415 | 6 | 89268715 | -0.09992 | 0.022733 | 0.0206 | 0.0365 | 19.32145 |
|  | 6 | rs3754624 | 2 | 225633812 | 0.097139 | 0.020006 | 0.03 | 0.0271 | 23.57482 |
|  | 7 | rs4278423 | 10 | 2670553 | 0.107529 | 0.023573 | 0.0055 | 0.0433 | 20.80846 |
|  | 8 | rs55876211 | 3 | 84471266 | -0.09119 | 0.01964 | -0.0036 | 0.0235 | 21.55693 |
|  | 9 | rs61933850 | 12 | 73139398 | 0.164808 | 0.036065 | 0.0748 | 0.0303 | 20.88295 |
|  | 10 | rs6679026 | 1 | 78619512 | 0.111984 | 0.025109 | 0.0228 | 0.0344 | 19.89116 |
|  | 11 | rs7001029 | 8 | 131958403 | 0.117366 | 0.026132 | 0.0185 | 0.0357 | 20.17093 |
|  | 12 | rs72714493 | 1 | 92206218 | 0.081686 | 0.018079 | -0.0014 | 0.0288 | 20.41454 |
|  | 13 | rs74354280 | 4 | 134192918 | -0.09119 | 0.020491 | 0.009 | 0.023 | 19.8051 |
|  | 14 | rs76784716 | 2 | 169033340 | 0.136049 | 0.028577 | 0.0175 | 0.0323 | 22.66557 |
|  | 15 | rs9813022 | 3 | 13726736 | -0.08424 | 0.016358 | 0.019 | 0.0213 | 26.52197 |
| genus Anaerostipes (ebi-a-GCST90016966) | 1 | rs10502061 | 11 | 105615253 | 0.083575 | 0.019202 | 0.0116 | 0.0324 | 18.94417 |
|  | 2 | rs2014785 | 3 | 171100102 | 0.051569 | 0.01122 | -0.0279 | 0.021 | 21.12489 |
|  | 3 | rs2396460 | 2 | 228018671 | 0.051265 | 0.010956 | -0.0065 | 0.0208 | 21.8969 |
|  | 4 | rs2804244 | 10 | 117383184 | 0.053089 | 0.011098 | 0.0228 | 0.0213 | 22.88212 |
|  | 5 | rs3900776 | 9 | 13525082 | -0.11001 | 0.02363 | 0.0904 | 0.063 | 21.67471 |
|  | 6 | rs60983350 | 17 | 2850737 | -0.05399 | 0.011658 | -0.0075 | 0.0223 | 21.44951 |
|  | 7 | rs62157625 | 2 | 142774333 | 0.088573 | 0.018555 | -0.0716 | 0.0316 | 22.78716 |
|  | 8 | rs62215703 | 21 | 25873624 | 0.064497 | 0.01367 | 0.0102 | 0.025 | 22.25987 |
|  | 9 | rs6474958 | 9 | 1582701 | 0.050007 | 0.0112 | -0.0045 | 0.0224 | 19.93467 |
|  | 10 | rs6726833 | 2 | 39351569 | -0.08775 | 0.018941 | -0.012 | 0.0386 | 21.46321 |
|  | 11 | rs6854026 | 4 | 169690814 | 0.050848 | 0.010908 | 0.0051 | 0.0208 | 21.73205 |
|  | 12 | rs7193624 | 16 | 77574020 | -0.07506 | 0.015072 | 0.0016 | 0.0373 | 24.80314 |
|  | 13 | rs78735375 | 19 | 1497547 | -0.13743 | 0.03053 | 0.136 | 0.0527 | 20.26244 |
| genus Anaerotruncus (ebi-a-GCST90016967) | 1 | rs10150232 | 14 | 30418008 | 0.056709 | 0.012488 | 0.0017 | 0.0258 | 20.62225 |
|  | 2 | rs11018566 | 11 | 89040226 | -0.15647 | 0.036603 | 3.00E-04 | 0.0458 | 18.27243 |
|  | 3 | rs115414803 | 4 | 88163243 | -0.14436 | 0.031752 | -0.0504 | 0.0428 | 20.66886 |
|  | 4 | rs1272208 | 9 | 78630894 | 0.061174 | 0.012983 | 0.0239 | 0.0244 | 22.20144 |
|  | 5 | rs1431492 | 3 | 150855371 | -0.0655 | 0.014619 | -0.0175 | 0.0283 | 20.07492 |
|  | 6 | rs17734739 | 2 | 211663702 | 0.066005 | 0.014908 | 0.0306 | 0.0296 | 19.60277 |
|  | 7 | rs34449434 | 12 | 76523655 | -0.0497 | 0.01134 | 0.0058 | 0.0218 | 19.20783 |
|  | 8 | rs4669806 | 2 | 12200752 | 0.057639 | 0.012299 | 0.042 | 0.0251 | 21.96158 |
|  | 9 | rs6494922 | 15 | 33459867 | 0.090311 | 0.020226 | 0.0954 | 0.046 | 19.93748 |
|  | 10 | rs6563550 | 13 | 38058413 | 0.087714 | 0.017675 | -0.0199 | 0.0383 | 24.62854 |
|  | 11 | rs7155595 | 14 | 77502546 | 0.053934 | 0.01189 | 0.0111 | 0.0225 | 20.57469 |
|  | 12 | rs8005030 | 14 | 30607199 | 0.055445 | 0.011785 | 0.0085 | 0.0219 | 22.13255 |
|  | 13 | rs9347879 | 6 | 165015261 | 0.050618 | 0.011049 | 0.0141 | 0.0206 | 20.98766 |
| genus Bacteroides (ebi-a-GCST90016968) | 1 | rs11585893 | 1 | 10644351 | -0.07407 | 0.014763 | -0.031 | 0.0242 | 25.17513 |
|  | 2 | rs13207588 | 6 | 41519430 | -0.05921 | 0.01312 | -0.006 | 0.0264 | 20.3649 |
|  | 3 | rs1340391 | 1 | 102960989 | -0.0592 | 0.013224 | -0.0596 | 0.0304 | 20.03998 |
|  | 4 | rs17619981 | 19 | 24342250 | 0.088098 | 0.0187 | 0.0098 | 0.0303 | 22.1941 |
|  | 5 | rs2023437 | 14 | 22045949 | -0.07823 | 0.016763 | 0.0034 | 0.0314 | 21.77962 |
|  | 6 | rs66474973 | 20 | 58605432 | 0.081252 | 0.016448 | 0.0401 | 0.0341 | 24.40292 |
|  | 7 | rs66710942 | 3 | 77215327 | -0.0488 | 0.010741 | -0.0043 | 0.0207 | 20.64437 |
|  | 8 | rs6795673 | 3 | 10593224 | 0.053857 | 0.010525 | 0.015 | 0.0206 | 26.18326 |
|  | 9 | rs9507307 | 13 | 24910476 | 0.060446 | 0.012913 | 0.0305 | 0.024 | 21.91233 |
| genus Bifidobacterium (ebi-a-GCST90016970) | 1 | rs12022129 | 1 | 207003374 | -0.06194 | 0.013894 | 0.0318 | 0.0231 | 19.87215 |
|  | 2 | rs182549 | 2 | 136616754 | -0.1197 | 0.012729 | 0.0153 | 0.021 | 88.4289 |
|  | 3 | rs2491158 | 10 | 126089703 | -0.07126 | 0.015983 | -0.0013 | 0.0306 | 19.87945 |
|  | 4 | rs2686790 | 7 | 48090746 | -0.07074 | 0.015793 | -1.00E-04 | 0.0291 | 20.06481 |
|  | 5 | rs4957061 | 5 | 521096 | 0.053424 | 0.011743 | -0.0018 | 0.021 | 20.69692 |
|  | 6 | rs540489 | 17 | 72897722 | -0.06376 | 0.013875 | 0.05 | 0.0271 | 21.12086 |
|  | 7 | rs55888705 | 4 | 1517826 | 0.054632 | 0.012114 | -0.0221 | 0.023 | 20.33877 |
|  | 8 | rs5746486 | 22 | 18354272 | -0.05362 | 0.01208 | -0.0162 | 0.0212 | 19.70328 |
|  | 9 | rs62181700 | 2 | 189805784 | -0.06246 | 0.013121 | 0.0045 | 0.0241 | 22.66538 |
|  | 10 | rs7322849 | 13 | 112859829 | 0.112428 | 0.020181 | -0.0833 | 0.0361 | 31.03492 |
|  | 11 | rs73797465 | 5 | 142793467 | -0.09536 | 0.020924 | 0.0199 | 0.0326 | 20.76963 |
|  | 12 | rs75344046 | 21 | 31861790 | 0.232354 | 0.050598 | -0.0773 | 0.0488 | 21.088 |
|  | 13 | rs857444 | 6 | 14617591 | 0.055823 | 0.012122 | -0.0153 | 0.0214 | 21.20758 |
| genus Christensenellaceae R7 (ebi-a-GCST90016978) | 1 | rs10461257 | 4 | 156131004 | -0.0552 | 0.012207 | 0.051 | 0.022 | 20.44648 |
|  | 2 | rs17081797 | 18 | 67555560 | -0.09043 | 0.020425 | 0.0032 | 0.0419 | 19.60253 |
|  | 3 | rs60954665 | 19 | 35883736 | 0.049815 | 0.011079 | -0.0243 | 0.0206 | 20.21821 |
|  | 4 | rs62132810 | 19 | 49279227 | -0.08289 | 0.017962 | 0.0152 | 0.0297 | 21.29272 |
|  | 5 | rs62190261 | 2 | 230943099 | 0.095839 | 0.021472 | 0.0117 | 0.0366 | 19.92191 |
|  | 6 | rs62467127 | 7 | 118096925 | 0.114108 | 0.025198 | 0.0257 | 0.0661 | 20.50641 |
|  | 7 | rs73952017 | 18 | 1779609 | -0.08622 | 0.019435 | 0.0235 | 0.034 | 19.67854 |
|  | 8 | rs78521377 | 10 | 126448374 | 0.124992 | 0.02748 | -0.104 | 0.0613 | 20.68905 |
|  | 9 | rs79150079 | 5 | 29352420 | 0.121547 | 0.027098 | 0.0104 | 0.0413 | 20.1198 |
|  | 10 | rs892686 | 9 | 83043376 | 0.05141 | 0.011136 | -0.0306 | 0.0207 | 21.31253 |
| genus Eubacterium hallii group (ebi-a-GCST90017000) | 1 | rs10501370 | 11 | 58040621 | -0.11559 | 0.025249 | 0.0749 | 0.0428 | 20.95792 |
|  | 2 | rs10798999 | 1 | 34308917 | 0.060166 | 0.012675 | 0.0551 | 0.0233 | 22.53171 |
|  | 3 | rs10808115 | 7 | 100635375 | 0.050475 | 0.010992 | 0.0136 | 0.0206 | 21.08523 |
|  | 4 | rs117748144 | 11 | 11771637 | -0.12658 | 0.028712 | -0.1166 | 0.0474 | 19.43645 |
|  | 5 | rs13116360 | 4 | 111885431 | 0.154124 | 0.029719 | 0.052 | 0.0417 | 26.89594 |
|  | 6 | rs138531890 | 3 | 195238086 | 0.153138 | 0.034946 | 0.0182 | 0.0384 | 19.20352 |
|  | 7 | rs17074066 | 4 | 183709536 | -0.0814 | 0.018927 | -0.0208 | 0.0751 | 18.49529 |
|  | 8 | rs17474256 | 1 | 104524676 | 0.081081 | 0.018457 | -0.0336 | 0.0349 | 19.2973 |
|  | 9 | rs281379 | 19 | 49214274 | -0.04995 | 0.011215 | 0.0016 | 0.021 | 19.8376 |
|  | 10 | rs28584818 | 3 | 64664456 | 0.126115 | 0.026863 | -0.0641 | 0.0384 | 22.04115 |
|  | 11 | rs60254196 | 7 | 148856720 | 0.052283 | 0.011187 | 6.00E-04 | 0.0207 | 21.84419 |
|  | 12 | rs630939 | 18 | 48384463 | -0.05089 | 0.011435 | -7.00E-04 | 0.0209 | 19.8062 |
|  | 13 | rs6550770 | 3 | 23663416 | 0.198087 | 0.044355 | 0.0554 | 0.0517 | 19.94512 |
|  | 14 | rs74018587 | 15 | 62014160 | 0.208943 | 0.043822 | 0.056 | 0.0523 | 22.73376 |
|  | 15 | rs78056098 | 11 | 123789877 | -0.05074 | 0.011376 | -0.0178 | 0.0215 | 19.89555 |
|  | 16 | rs949971 | 3 | 110283389 | -0.05402 | 0.01161 | 0.0497 | 0.022 | 21.64608 |
| Genus Lachnospiraceae UCG001(ebi-a-GCST90017025) | 1 | rs12131224 | 1 | 166199362 | 0.117095 | 0.02591 | 0.0293 | 0.1088 | 20.42405 |
|  | 2 | rs2050911 | 1 | 82384407 | 0.0751466 | 0.0153934 | -0.0105 | 0.3379 | 23.8314 |
|  | 3 | rs2371284 | 12 | 56256262 | 0.0761893 | 0.0170127 | 0.0654 | 0.7678 | 20.05587 |
|  | 4 | rs437876 | 3 | 42568440 | 0.0784637 | 0.0144768 | -0.0077 | 0.3552 | 29.37598 |
|  | 5 | rs4981345 | 14 | 21455973 | -0.0681973 | 0.0149831 | 0.0084 | 0.3296 | 20.7172 |
|  | 6 | rs573933 | 9 | 14477851 | -0.107898 | 0.0232346 | -0.0127 | 0.1065 | 21.56534 |
|  | 7 | rs62496417 | 7 | 96965504 | -0.0748504 | 0.0165681 | -0.0277 | 0.2149 | 20.40999 |
|  | 8 | rs7341608 | 8 | 56839500 | -0.0784757 | 0.0177648 | -0.0104 | 0.1341 | 19.51416 |
|  | 9 | rs74034332 | 16 | 79092359 | 0.168045 | 0.0382637 | 0.0265 | 0.06167 | 19.28756 |
|  | 10 | rs78848836 | 1 | 53773248 | -0.118868 | 0.0259753 | 0.0152 | 0.1038 | 20.94155 |
|  | 11 | rs8104225 | 19 | 13962924 | 0.0892098 | 0.0197665 | 0.039 | 0.2276 | 20.36881 |
|  | 12 | rs9403580 | 6 | 145006780 | 0.107801 | 0.0229777 | 0.0535 | 0.1288 | 22.01063 |
|  | 13 | rs985416 | 3 | 148269083 | -0.0970258 | 0.0181807 | -0.0029 | 0.8199 | 28.48087 |
| Unknown genus(ebi-a-GCST90017084) | 1 | rs10200320 | 2 | 197967710 | -0.06411 | 0.014277 | 0.008 | 0.0255 | 20.16678 |
|  | 2 | rs11195523 | 10 | 113086067 | -0.06893 | 0.014535 | -0.0216 | 0.0238 | 22.4881 |
|  | 3 | rs11684166 | 2 | 111817641 | -0.07696 | 0.016835 | -0.0528 | 0.027 | 20.90011 |
|  | 4 | rs11809762 | 1 | 175159585 | -0.09346 | 0.01902 | -0.0568 | 0.0262 | 24.14745 |
|  | 5 | rs11904514 | 2 | 26541158 | 0.109498 | 0.024984 | -0.0493 | 0.0427 | 19.20843 |
|  | 6 | rs12147596 | 14 | 104006716 | -0.07198 | 0.014161 | 0.0096 | 0.0222 | 25.83829 |
|  | 7 | rs16823675 | 2 | 145173029 | -0.07675 | 0.014997 | -0.0254 | 0.0256 | 26.19073 |
|  | 8 | rs17086536 | 6 | 124369748 | -0.10085 | 0.022432 | -0.0634 | 0.0395 | 20.2127 |
|  | 9 | rs2939766 | 11 | 41417982 | -0.05916 | 0.013042 | -0.0118 | 0.0207 | 20.5771 |
|  | 10 | rs34985298 | 7 | 93746275 | -0.06235 | 0.013772 | 0.0108 | 0.0219 | 20.49817 |
|  | 11 | rs35740166 | 4 | 105068883 | -0.11225 | 0.022682 | 0.0513 | 0.0351 | 24.48859 |
|  | 12 | rs4644504 | 1 | 221985829 | -0.09693 | 0.021615 | -0.0568 | 0.0411 | 20.11133 |
|  | 13 | rs6007642 | 22 | 47653347 | -0.07914 | 0.017796 | -0.0627 | 0.0248 | 19.77745 |
|  | 14 | rs72700702 | 15 | 35278445 | -0.09173 | 0.018901 | -0.0058 | 0.0329 | 23.55257 |
|  | 15 | rs76532867 | 12 | 94212918 | 0.112353 | 0.024236 | 0.0506 | 0.0536 | 21.48984 |
| Order Bifidobacteriales (ebi-a-GCST90017093) | 1 | rs10831953 | 11 | 13098051 | 0.053754 | 0.012375 | -0.0124 | 0.0225 | 18.86924 |
|  | 2 | rs12446429 | 16 | 898055 | 0.08101 | 0.019073 | 0.0179 | 0.0267 | 18.04007 |
|  | 3 | rs13020688 | 2 | 192878532 | 0.058405 | 0.012208 | -0.0464 | 0.0226 | 22.8874 |
|  | 4 | rs182549 | 2 | 136616754 | -0.11707 | 0.01267 | 0.0153 | 0.021 | 85.37239 |
|  | 5 | rs4957061 | 5 | 521096 | 0.056987 | 0.011691 | -0.0018 | 0.021 | 23.76157 |
|  | 6 | rs540489 | 17 | 72897722 | -0.06325 | 0.013817 | 0.05 | 0.0271 | 20.95648 |
|  | 7 | rs55888705 | 4 | 1517826 | 0.053673 | 0.012059 | -0.0221 | 0.023 | 19.81153 |
|  | 8 | rs6899771 | 6 | 97406220 | -0.09143 | 0.02026 | 0.0303 | 0.0342 | 20.36539 |
|  | 9 | rs7174549 | 15 | 92463303 | -0.05517 | 0.012466 | -0.0331 | 0.0215 | 19.58969 |
|  | 10 | rs7322849 | 13 | 112859829 | 0.110676 | 0.0201 | -0.0833 | 0.0361 | 30.3202 |
|  | 11 | rs73797465 | 5 | 142793467 | -0.09426 | 0.020842 | 0.0199 | 0.0326 | 20.45422 |
|  | 12 | rs857444 | 6 | 14617591 | 0.055397 | 0.012067 | -0.0153 | 0.0214 | 21.07527 |
| Phylum Tenericutes (ebi-a-GCST90017117) | 1 | rs10108398 | 8 | 59440824 | 0.076914 | 0.015395 | 0.032 | 0.0231 | 24.95955 |
|  | 2 | rs11890098 | 2 | 157532549 | 0.074438 | 0.015339 | 0.0012 | 0.023 | 23.55054 |
|  | 3 | rs12566890 | 1 | 61850864 | -0.10115 | 0.023098 | 0.0271 | 0.0308 | 19.1763 |
|  | 4 | rs17214486 | 14 | 97159674 | 0.06099 | 0.013562 | -0.0214 | 0.0222 | 20.22323 |
|  | 5 | rs2464826 | 7 | 79490250 | 0.094424 | 0.021181 | -0.017 | 0.0327 | 19.87409 |
|  | 6 | rs28537087 | 15 | 95309676 | 0.082087 | 0.018831 | -0.0565 | 0.024 | 19.00185 |
|  | 7 | rs3768491 | 1 | 109965986 | 0.068105 | 0.014906 | -0.0316 | 0.0227 | 20.87529 |
|  | 8 | rs4885016 | 13 | 73170489 | 0.081961 | 0.018163 | -0.0204 | 0.0301 | 20.36314 |
|  | 9 | rs6043847 | 20 | 16259524 | -0.11494 | 0.024861 | 0.0419 | 0.0439 | 21.37453 |
|  | 10 | rs72901605 | 11 | 47103877 | -0.08419 | 0.017812 | -0.0368 | 0.0327 | 22.33835 |
|  | 11 | rs74603314 | 14 | 46519718 | 0.221639 | 0.046292 | -0.0454 | 0.0531 | 22.92367 |
|  | 12 | rs78169027 | 11 | 108439087 | -0.10828 | 0.023729 | 0.0428 | 0.0438 | 20.82406 |
