# Supplementary material for: Gut microbiota and atopic dermatitis: a two-sample Mendelian randomization study
Source: Front Med (Lausanne). 2023 Jun 22;10:1174331. doi: 10.3389/fmed.2023.1174331 (PMC10323683; doi:10.3389/fmed.2023.1174331)
Supplement: Supplementary file 3 [file Table_3.DOCX]

**Supplementary Table 3. SNPs were used as instrumental variables about gut microbiome abundance and AD GWASs (*P*<5×10^-8^).**

| Bacterial traits | SNP | CHR | Position | Effect allele | Other allele | Gut microbiota | | | AD | | | Proxy SNP | Target effect allele | Target other allele | F |
| --- | --- | --- | --- | --- | --- | --- | --- | --- | --- | --- | --- | --- | --- | --- | --- |
|  |  |  |  |  |  | Beta | SE | P-val | Beta | SE | P-val |  |  |  |  |
| Actinobacteria | **rs182549** | 2 | 136616754 | T | C | -0.111 | 0.012 | 3.79E-20 | 0.015 | 0.021 | 0.466 |  |  |  | 30.803 |
| Bifidobacteriaceae | rs182549 | 2 | 136616754 | T | C | -0.117 | 0.013 | 5.94E-20 | 0.015 | 0.021 | 0.466 |  |  |  | 36.572 |
|  | **rs7322849** | 13 | 112859829 | T | C | 0.111 | 0.020 | 1.74E-08 | -0.083 | 0.036 | 0.021 |  |  |  | 33.387 |
| Oxalobacteraceae | **rs4428215** | 3 | 171947435 | G | A | 0.126 | 0.023 | 4.88E-08 | -0.011 | 0.024 | 0.632 |  |  |  | 31.194 |
| Peptostreptococcaceae | **rs61841503** | 10 | 17019559 | G | A | 0.092 | 0.016 | 9.80E-09 | -0.026 | 0.031 | 0.399 |  |  |  | 32.654 |
| Streptococcaceae | **rs11110281** | 12 | 100584014 | T | C | -0.131 | 0.023 | 1.40E-08 | -0.009 | 0.049 | 0.851 |  |  |  | 85.376 |
| unknown family | **rs9864379** | 3 | 14306949 | T | C | -0.161 | 0.029 | 4.66E-08 | -0.066 | 0.029 | 0.023 |  |  |  | 85.372 |
| Allisonella | **rs602075** | 9 | 79110160 | G | A | -0.169 | 0.030 | 3.57E-08 | 0.021 | 0.023 | 0.380 |  |  |  | 30.320 |
| Bifidobacterium | rs182549 | 2 | 136616754 | T | C | -0.119 | 0.013 | 1.28E-20 | 0.015 | 0.021 | 0.466 |  |  |  | 85.372 |
|  | rs7322849 | 13 | 112859829 | T | C | 0.112 | 0.020 | 1.08E-08 | -0.083 | 0.036 | 0.021 |  |  |  | 30.320 |
| Candidatus Soleaferrea | **rs830151** | 19 | 47985753 | A | G | -0.195 | 0.035 | 4.26E-08 | 0.019 | 0.044 | 0.656 | **rs830147** | A | G | 88.429 |
| Erysipelatoclostridium | **rs7221249** | 17 | 10177708 | G | A | -0.084 | 0.014 | 4.31E-09 | -0.004 | 0.021 | 0.845 |  |  |  | 31.035 |
| Eubacterium coprostanoligenes group | **rs17159861** | 7 | 31085162 | C | T | 0.096 | 0.017 | 1.04E-08 | -0.020 | 0.034 | 0.546 |  |  |  | 31.285 |
| Intestinibacter | **rs10805326** | 4 | 14324623 | A | G | -0.078 | 0.014 | 3.55E-08 | -0.009 | 0.023 | 0.687 |  |  |  | 29.812 |
| Oxalobacter | **rs736744** | 9 | 87514407 | T | C | -0.118 | 0.021 | 2.57E-08 | 0.015 | 0.021 | 0.488 |  |  |  | 32.374 |
| Romboutsia | rs61841503 | 10 | 17019559 | G | A | 0.093 | 0.017 | 4.00E-08 | -0.026 | 0.031 | 0.399 |  |  |  | 29.351 |
| Ruminococcaceae UCG013 | **rs12781711** | 10 | 2219930 | C | T | -0.066 | 0.012 | 2.55E-08 | 0.044 | 0.024 | 0.067 |  |  |  | 32.495 |
| Ruminococcus torques group | **rs35866622** | 19 | 49218060 | T | C | -0.061 | 0.011 | 2.21E-08 | 0.022 | 0.022 | 0.325 |  |  |  | 35.417 |
| Streptococcus | rs11110281 | 12 | 100584014 | T | C | -0.138 | 0.023 | 2.58E-09 | -0.138 | 0.049 | 0.851 |  |  |  | 34.619 |
| Tyzzerella3 | **rs67476743** | 19 | 1030320 | T | G | 0.132 | 0.022 | 3.74E-09 | -0.046 | 0.023 | 0.050 |  |  |  | 31.135 |
| unknown genus | rs9864379 | 3 | 14306949 | T | C | -0.161 | 0.029 | 4.66E-08 | -0.066 | 0.029 | 0.023 |  |  |  | 58.161 |
| Bifidobacteriales | rs182549 | 2 | 136616754 | T | C | -0.117 | 0.013 | 5.94E-20 | 0.015 | 0.021 | 0.466 |  |  |  | 31.270 |
|  | rs7322849 | 13 | 112859829 | T | C | 0.111 | 0.020 | 1.74E-08 | -0.083 | 0.036 | 0.021 |  |  |  | 30.065 |
| Gastranaerophilales | rs9864379 | 3 | 14306949 | T | C | -0.161 | 0.029 | 4.66E-08 | -0.066 | 0.029 | 0.023 |  |  |  | 30.065 |
| Actinobacteria | **rs7570971** | 2 | 135837906 | A | C | 0.087 | 0.011 | 1.41E-14 | -0.016 | 0.021 | 0.436 |  |  |  | 30.065 |
