# Supplementary material for: Gut microbiota and atopic dermatitis: a two-sample Mendelian randomization study
Source: Front Med (Lausanne). 2023 Jun 22;10:1174331. doi: 10.3389/fmed.2023.1174331 (PMC10323683; doi:10.3389/fmed.2023.1174331)
Supplement: Supplementary file 4 [file Table_4.DOCX]

Supplementary Table 4. MR results of causal links between the gut microbiome and AD risk (P <1 × 10^-5^).

| Classification | |  | Nsnp | Methods | Beta | SE | OR (95% CI) | P-value | Horizontal pleiotropy | | | Heterogeneity | |
| --- | --- | --- | --- | --- | --- | --- | --- | --- | --- | --- | --- | --- | --- |
|  |  |  |  |  |  |  |  |  | Egger  Intercept | SE | P-value | Cochran’s  Q methods | P-value |
| phylum | Tenericutes id.3919 | | 12 | MR Egger  WME  IVW  Simple mode  Weighted mode | -0.252  -0.240  -0.193  -0.302  -0.262 | 0.342  0.130  0.096  0.218  0.206 | 0.778(0.398,1.520)  0.787(0.609,1.015)  0.824(0.683,0.995)  0.739(0.482,1.134)  0.770(0.514,2.153) | 0.479  0.065  0.044  0.194  0.231 | 0.005 | 0.030 | 0.862 | MR Egger  IVW | 0.325  0.406 |
| class | Mollicutes id.3920 | | 12 | MR Egger  WME  IVW  Simple mode  Weighted mode | -0.252  -0.240  -0.193  -0.302  -0.262 | 0.342  0.130  0.096  0.224  0.207 | 0.778(0.398,1.520)  0.787(0.609,1.015)  0.824(0.683,0.995)  0.739(0.482,1.134)  0.770(0.513,1.154) | 0.479  0.065  0.044  0.204  0.232 | 0.005 | 0.030 | 0.862 | MR Egger  IVW | 0.326  0.407 |
| class | Clostridia id.1859 | | 12 | MR Egger  WME  IVW  Simple mode  Weighted mode | -0.359  -0.280  -0.230  -0.403  -0.395 | 0.300  0.153  0.111  0.278  0.273 | 0.698(0.388,1.258)  0.756(0.560,1.019)  0.794(0.639,0.987)  0.668(0.397,1.153)  0.674(0.395,1.150) | 0.259  0.067  0.037  0.175  0.175 | 0.010 | 0.021 | 0.665 | MR Egger  IVW | 0.603  0.670 |
| class | Bacilli id.1673 | | 18 | MR Egger  WME  IVW  Simple mode  Weighted mode | 0.586  0.098  0.056  0.137  0.142 | 0.255  0.128  0.093  0.269  0.242 | 0.556(0.337,0.918)  1.103(0.858,1.418)  1.057(0.882,1.268)  1.147(0.677,1.944)  1.153(0.717,1.852) | 0.036  0.443  0.547  0.617  0.565 | 0.051 | 0.019 | 0.016 | MR Egger  IVW | 0.966  0.623 |
| family | Clostridiaceae_1 id.1869 | | 10 | MR Egger  WME  IVW  Simple mode  Weighted mode | 0.446  0.246  0.229  0.188  0.269 | 0.332  0.160  0.114  0.256  0.247 | 1.563(0.816,2.994)  1.278(0.934,1.749)  1.257(1.007,1.571)  1.207(0.730,1.994)  1.308(0.807,2.122) | 0.215  0.125  0.044  0.482  0.304 | -0.015 | 0.024 | 0.536 | MR Egger  IVW | 0.693  0.738 |
| family | Bifidobacteriaceae id.433 | | 12 | MR Egger  WME  IVW  Simple mode  Weighted mode | -0.277  -0.189  -0.225  -0.240  -0.177 | 0.368  0.140  0.107  0.224  0.156 | 0.758(0.368,1.558)  0.827(0.629,1.088)  0.799(0.648,0.984)  0.786(0.507,1.219)  0.838(0.617,1.138) | 0.468  0.175  0.035  0.305  0.282 | 0.009 | 0.023 | 0.708 | MR Egger  IVW | 0.283  0.334 |
| family | Rhodospirillaceae id.2717 | | 15 | MR Egger  WME  IVW  Simple mode  Weighted mode | 0.772  0.044  0.065  0.082  -0.137 | 0.303  0.104  0.072  0.188  0.194 | 2.163(1.194,3.920)  1.045(0.852,1.282)  1.068(0.928,1.228)  1.085(0.751,1.568)  0.872(0.597,1.275) | 0.024  0.675  0.360  0.670  0.492 | -0.072 | 0.030 | 0.032 | MR Egger  IVW | 0.986  0.749 |
| family | Bacteroidaceae id.917 | | 9 | MR Egger  WME  IVW  Simple mode  Weighted mode | 0.180  0.257  0.302  0.150  0.173 | 0.686  0.165  0.132  0.249  0.252 | 1.197(0.312,4.589)  1.293(0.936,1.788)  1.353(1.044,1.753)  1.161(0.713,1.891)  1.189(0.726,1.949) | 0.801  0.119  0.022  0.565  0.511 | 0.027 | 0.040 | 0.518 | MR Egger  IVW | 0.881  0.899 |
| order | Bifidobacteriales id.432 | | 12 | MR Egger  WME  IVW  Simple mode  Weighted mode | -0.277  -0.189  -0.225  -0.240  -0.177 | 0.368  0.133  0.107  0.243  0.169 | 0.758(0.368,1.558)  0.827(0.637,1.075)  0.799(0.648,0.984)  0.786(0.4899,1.265)  0.838(0.602,1.166) | 0.468  0.156  0.035  0.343  0.317 | 0.004 | 0.027 | 0.884 | MR Egger  IVW | 0.190  0.251 |
| genus | Bifidobacterium id.436 | | 13 | MR Egger  WME  IVW  Simple mode  Weighted mode | -0.361  -0.187  -0.243  -0.128  -0.168 | 0.219  0.117  0.088  0.181  0.150 | 0.697(0.454,1.071)  0.830(0.659,1.044)  0.784(0.660,0.931)  0.880(0.617,1.254)  0.846(0.630,1.135) | 0.128  0.111  0.006  0.492  0.286 | 0.010 | 0.018 | 0.570 | MR Egger  IVW | 0.705  0.750 |
| genus | Bacteroides id.918 | | 9 | MR Egger  WME  IVW  Simple mode  Weighted mode | 0.180  0.257  0.302  0.150  0.173 | 0.686  0.175  0.132  0.233  0.230 | 1.197(0.312,4.589)  1.293(0.918,1.822)  1.353(1.044,1.753)  1.161(0.736,1.832)  1.189(0.758,1.867) | 0.801  0.141  0.022  0.539  0.473 | 0.008 | 0.044 | 0.860 | MR Egger  IVW | 0.785  0.858 |
| genus | Anaerotruncus id.2054 | | 13 | MR Egger  WME  IVW  Simple mode  Weighted mode | 0.210  0.246  0.246  0.251  0.247 | 0.321  0.149  0.110  0.234  0.210 | 1.234(0.657,2.317)  1.279(0.955,1.712)  1.279(1.031,1.588)  1.286(0.813,2.035)  1.280(0.849,1.932) | 0.526  0.098  0.026  0.304  0.262 | 0.003 | 0.022 | 0.908 | MR Egger  IVW | 0.790  0.850 |
| genus | unknown genus id.2071 | | 15 | MR Egger  WME  IVW  Simple mode  Weighted mode | 0.244  0.263  0.222  0.514  0.495 | 0.570  0.142  0.109  0.295  0.295 | 1.276(0.417,3.901)  1.301(0.985,1.718)  1.249(1.008,1.546)  1.672(0.939,2.981)  1.640(0.919,2.926) | 0.676  0.064  0.042  0.103  0.116 | -0.002 | 0.045 | 0.969 | MR Egger  IVW | 0.085  0.118 |
| genus | Lachnospiraceae UCG001 id.11321 | | 13 | MR Egger  WME  IVW  Simple mode  Weighted mode | 0.206  0.128  0.172  0.039  0.024 | 0.363  0.111  0.082  0.186  0.181 | 1.228(0.602,2.504)  1.137(0.914,1.413)  1.188(1.012,1.395)  1.040(0.722,1.497)  1.024(0.718,1.460) | 0.583  0.248  0.036  0.837  0.898 | -0.003 | 0.033 | 0.927 | MR Egger  IVW | 0.403  0.486 |
| genus | Eubacterium hallii group id.11338 | | 16 | MR Egger  WME  IVW  Simple mode  Weighted mode | 0.208  0.259  0.100  0.252  0.252 | 0.247  0.126  0.117  0.210  0.162 | 1.231(0.759,1.997)  1.295(1.012,1.659)  1.105(0.879,1.390)  1.286(0.853,1.940)  1.286(0.936,1.769) | 0.415  0.040  0.391  0.248  0.142 | -0.010 | 0.020 | 0.627 | MR Egger  IVW | 0.019  0.025 |
| genus | Christensenellaceae R 7group id.11283 | | 10 | MR Egger  WME  IVW  Simple mode  Weighted mode | 0.414  -0.202  -0.282  -0.030  -0.050 | 0.402  0.171  0.129  0.298  0.266 | 1.513(0.687,3.329)  0.817(0.584,1.143)  0.754(0.585,0.971)  0.971(0.542,1.739)  0.952(0.565,1.603) | 0.334  0.238  0.029  0.922  0.856 | -0.054 | 0.029 | 0.105 | MR Egger  IVW | 0.780  0.522 |
| genus | Anaerostipes id.1991 | | 12 | MR Egger  WME  IVW  Simple mode  Weighted mode | -1.064  0.041  -0.178  0.094  0.107 | 0.418  0.165  0.129  0.276  0.255 | 0.345(0.152,0.782)  1.042(0.754,1.441)  0.837(0.650,1.078)  1.099(0.640,1.888)  1.113(0.676,1.833) | 0.027  0.802  0.169  0.738  0.681 | 0.059 | 0.027 | 0.049 | MR Egger  IVW | 0.511  0.237 |
