# Supplementary material for: Gut microbiota and atopic dermatitis: a two-sample Mendelian randomization study
Source: Front Med (Lausanne). 2023 Jun 22;10:1174331. doi: 10.3389/fmed.2023.1174331 (PMC10323683; doi:10.3389/fmed.2023.1174331)
Supplement: Supplementary file 5 [file Table_5.DOCX]

- **Supplementary Table 5. The table of MR Analysis of gut microbiota and AD obtained at conventional thresholds (5×10^-8^).**

| N | Gut microbiota | Method | Nsnp | Beta | SE | OR | P-value |
| --- | --- | --- | --- | --- | --- | --- | --- |
| 1 | class Actinobacteria | Wald ratio | 1 | -0.137 | 0.188 | 0.872（0.603，1.261） | 0.466 |
| 2 | family Bifidobacteriaceae | Inverse variance weighted | 2 | -0.275 | 0.263 | 0.759（0.454，1.271） | 0.295 |
| 3 | family Oxalobacteraceae | Wald ratio | 1 | -0.090 | 0.189 | 0.914（0.631，1.323） | 0.634 |
| 4 | family Peptostreptococcaceae | Wald ratio | 1 | -0.282 | 0.335 | 0.755（0.391，1.455） | 0.400 |
| 5 | family Streptococcaceae | Wald ratio | 1 | 0.070 | 0.372 | 1.073（0.517，2.226） | 0.850 |
| **6** | **unknown family** | **Wald ratio** | **1** | **0.411** | **0.181** | 1.509（1.057，2.152） | **0.023** |
| 7 | genus Allisonella | Wald ratio | 1 | -0.122 | 0.138 | 0.885（0.675，1.161） | 0.379 |
| 8 | genus Bifidobacterium | Inverse variance weighted | 2 | -0.269 | 0.258 | 0.764（0.461，1.267） | 0.297 |
| 9 | genus Candidatus Soleaferrea | Wald ratio | 1 | -0.099 | 0.224 | 0.906（0.584，1.404） | 0.657 |
| 10 | genus Erysipelatoclostridium | Wald ratio | 1 | 0.048 | 0.245 | 1.049（0.648，1.696） | 0.846 |
| 11 | genus Eubacterium coprostanoligenes group | Wald ratio | 1 | -0.211 | 0.349 | 0.810（0.408，1.606） | 0.546 |
| 12 | genus Intestinibacter | Wald ratio | 1 | 0.117 | 0.293 | 1.125（0.633，1.996） | 0.689 |
| 13 | genus Oxalobacter | Wald ratio | 1 | -0.123 | 0.176 | 0.884（0.626，1.250） | 0.486 |
| 14 | genus Romboutsia | Wald ratio | 1 | -0.279 | 0.332 | 0.757（0.395，1.449） | 0.400 |
| 15 | genus Ruminococcaceae UCG013 | Wald ratio | 1 | -0.666 | 0.363 | 0.514（0.252，1.046） | 0.066 |
| 16 | genus Ruminococcus torques group | Wald ratio | 1 | -0.351 | 0.356 | 0.704（0.350，1.415） | 0.324 |
| 17 | genus Streptococcus | Wald ratio | 1 | 0.067 | 0.353 | 1.069（0.535，2.137） | 0.850 |
| 18 | genus Tyzzerella3 | Wald ratio | 1 | -0.347 | 0.177 | 0.707（0.500，1.001） | 0.050 |
| 19 | **unknown genus** | **Wald ratio** | **1** | **0.411** | **0.181** | 1.509（1.057，2.152） | **0.023** |
| 20 | order Bifidobacteriales | Inverse variance weighted | 2 | -0.275 | 0.263 | 0.760（0.454，1.271） | 0.295 |
| **21** | **order Gastranaerophilales** | **Wald ratio** | **1** | **0.411** | **0.181** | 1.509（1.057，2.152） | **0.023** |
| 22 | phylum Actinobacteria | Wald ratio | 1 | -0.188 | 0.241 | 0.829（0.516，1.329） | 0.435 |
