# Supplementary figures and images for: Gut microbiota and atopic dermatitis: a two-sample Mendelian randomization study
Source: Front Med (Lausanne). 2023 Jun 22;10:1174331. doi: 10.3389/fmed.2023.1174331 (PMC10323683; doi:10.3389/fmed.2023.1174331)

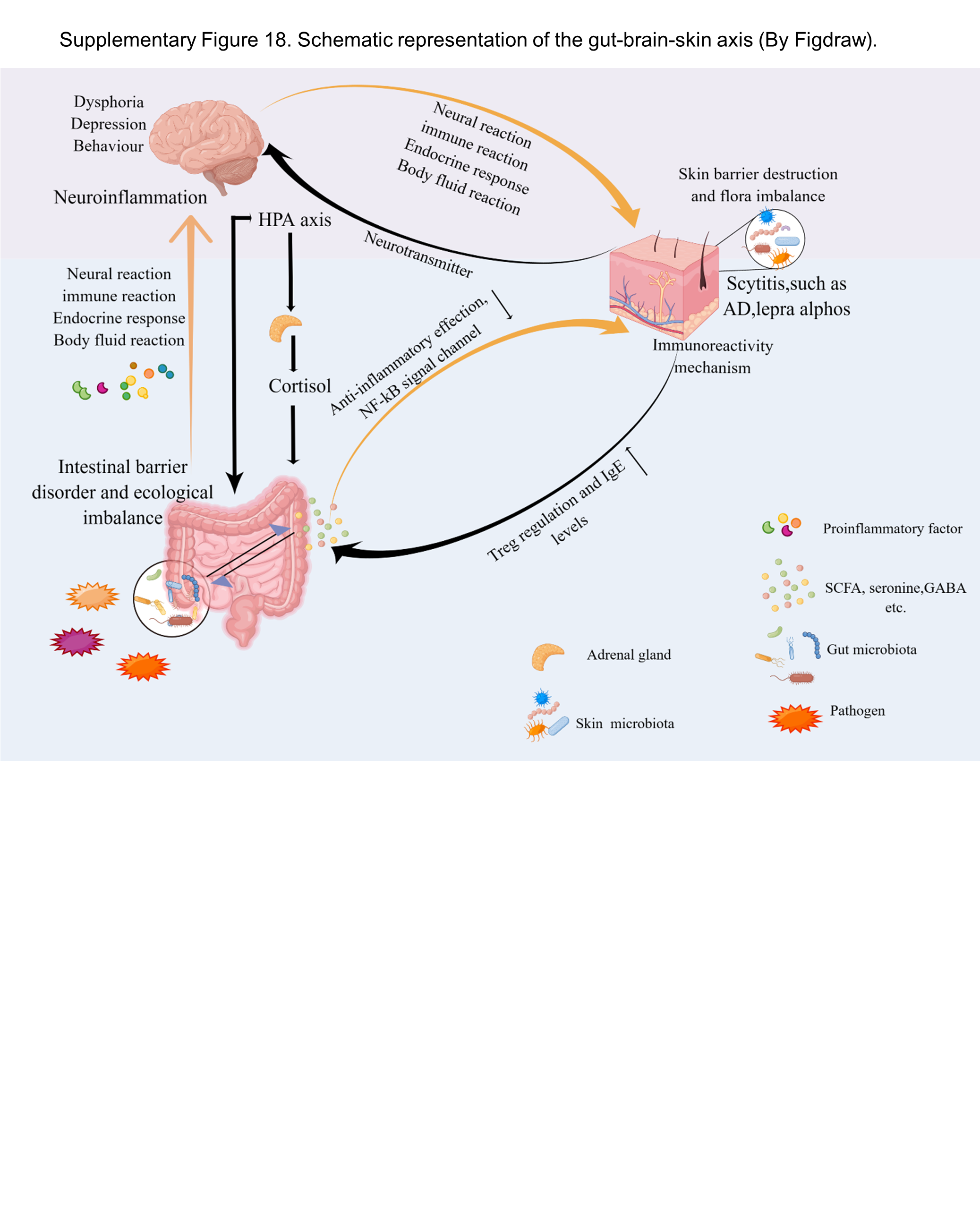

Supplement: Supplementary file 6 [file Data_Sheet_1.ZIP › Supplementary Figure 18.tif]

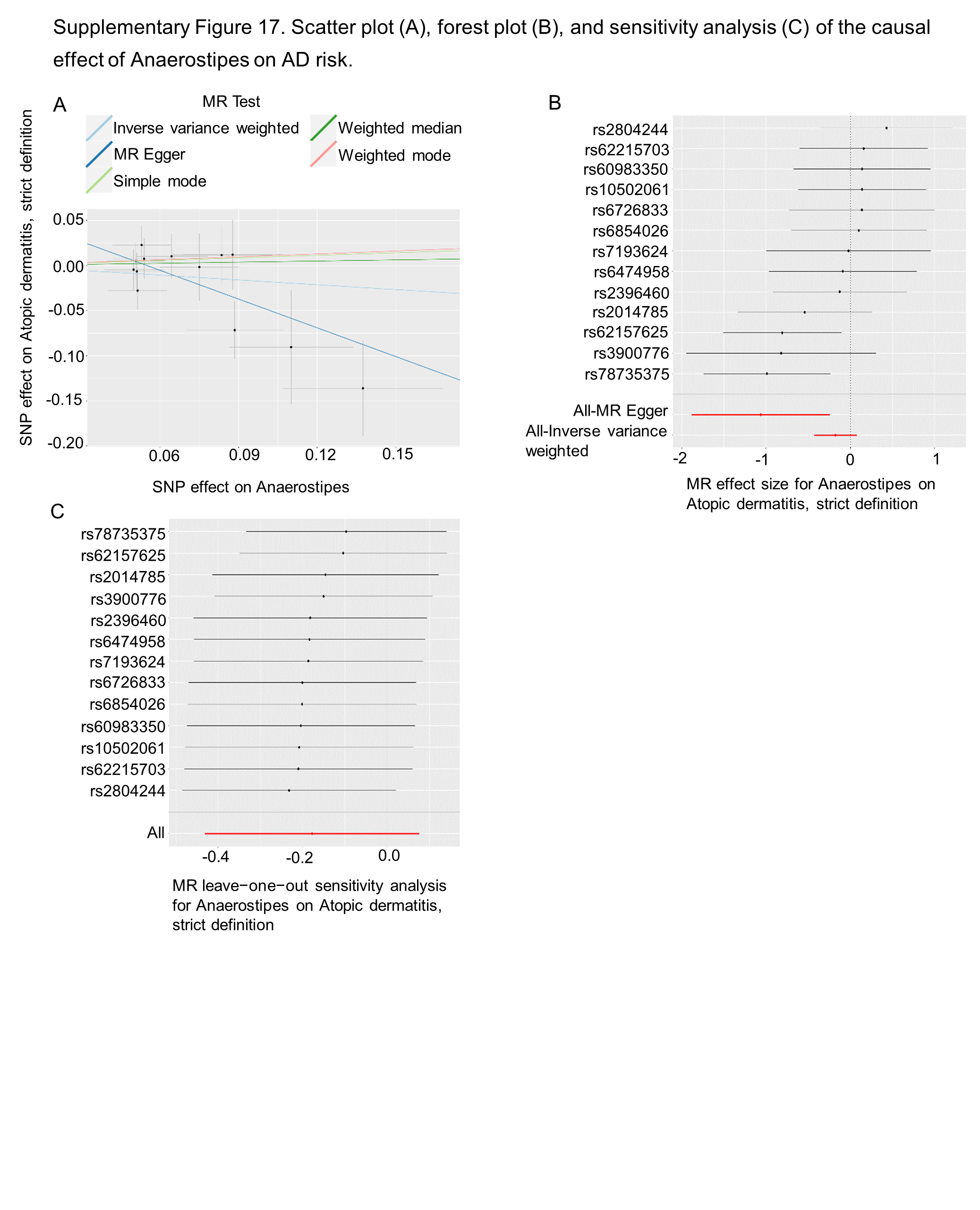

Supplement: Supplementary file 6 [file Data_Sheet_1.ZIP › Supplementary Figure 17.tif]

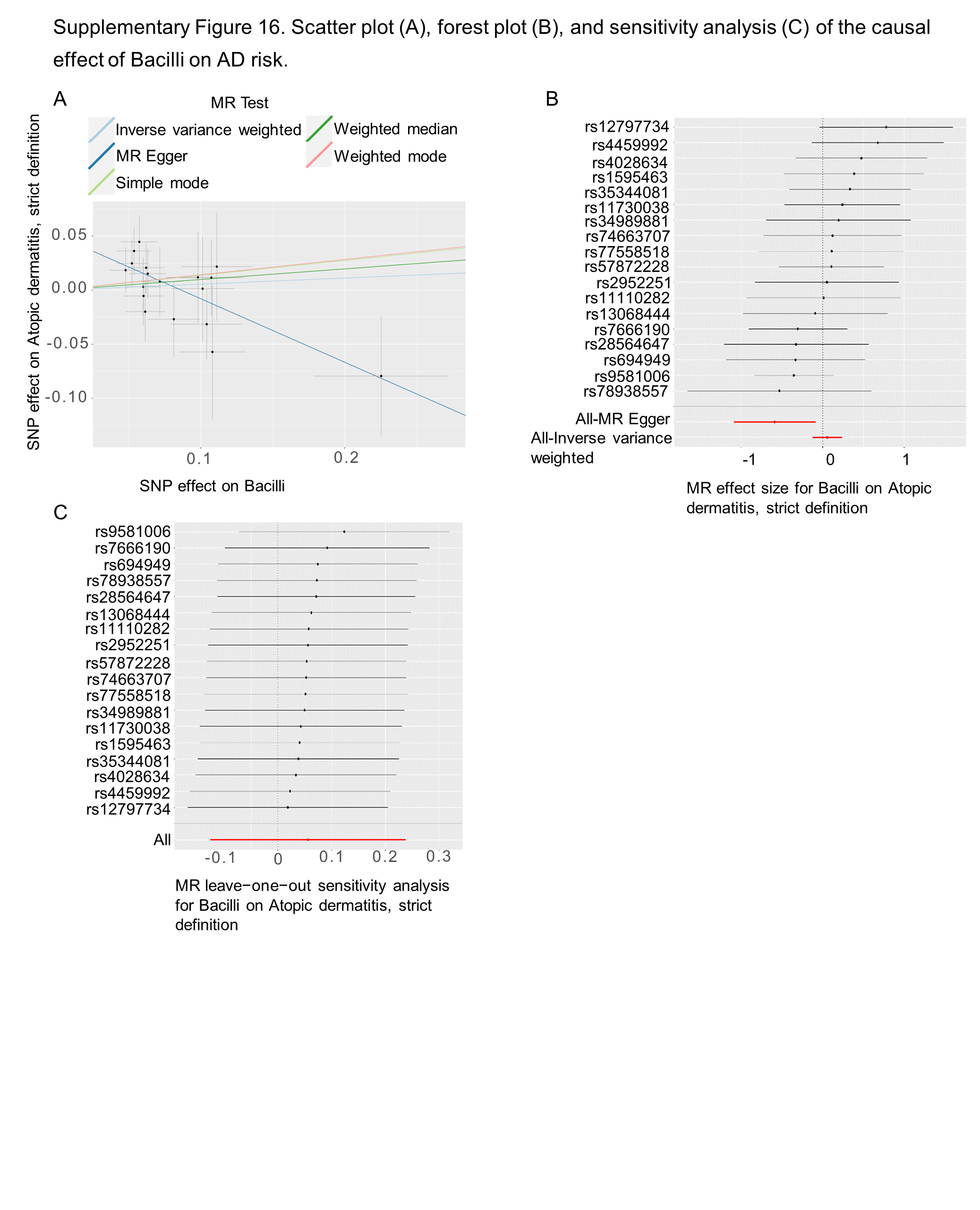

Supplement: Supplementary file 6 [file Data_Sheet_1.ZIP › Supplementary Figure 16.tif]

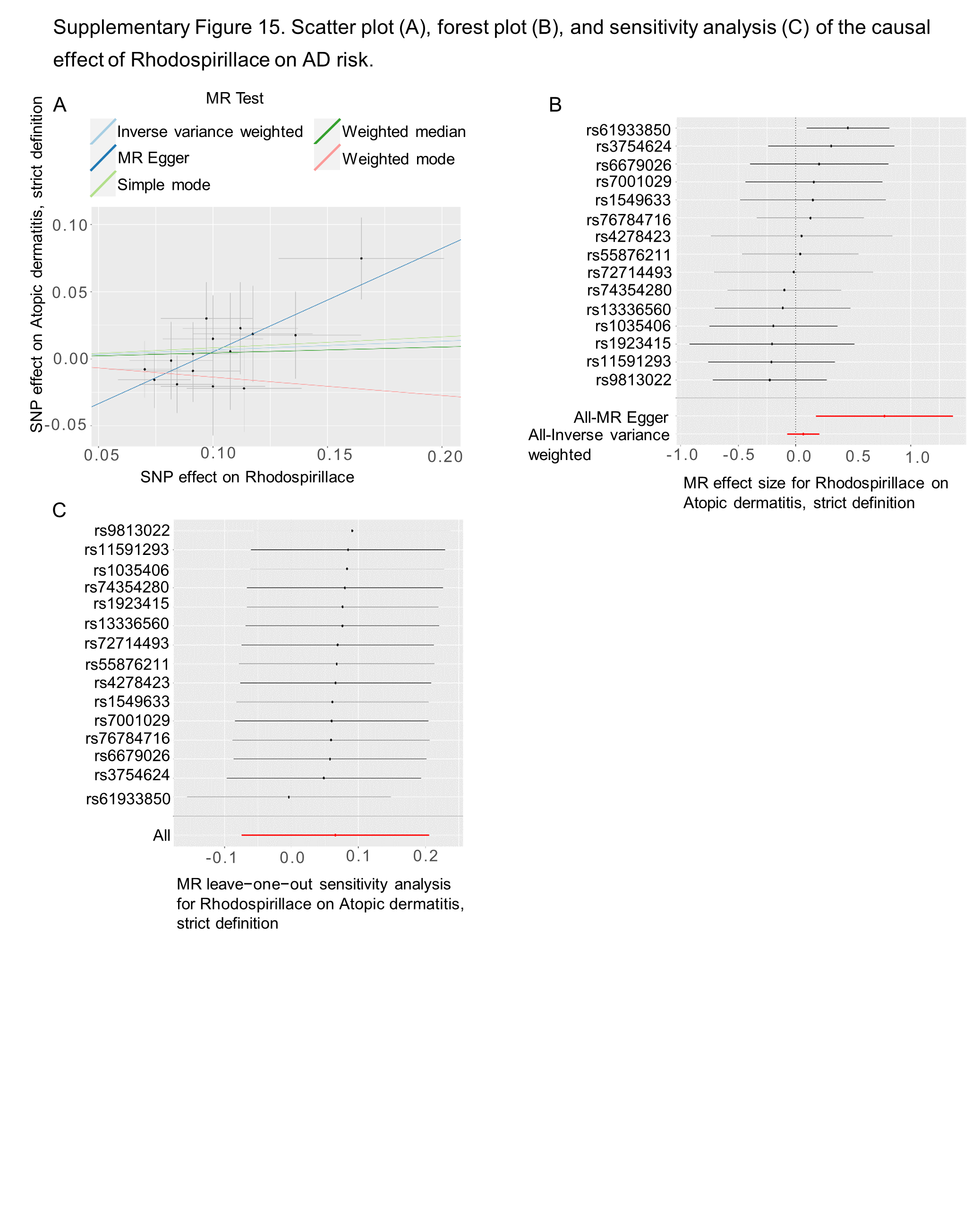

Supplement: Supplementary file 6 [file Data_Sheet_1.ZIP › Supplementary Figure 15.tif]

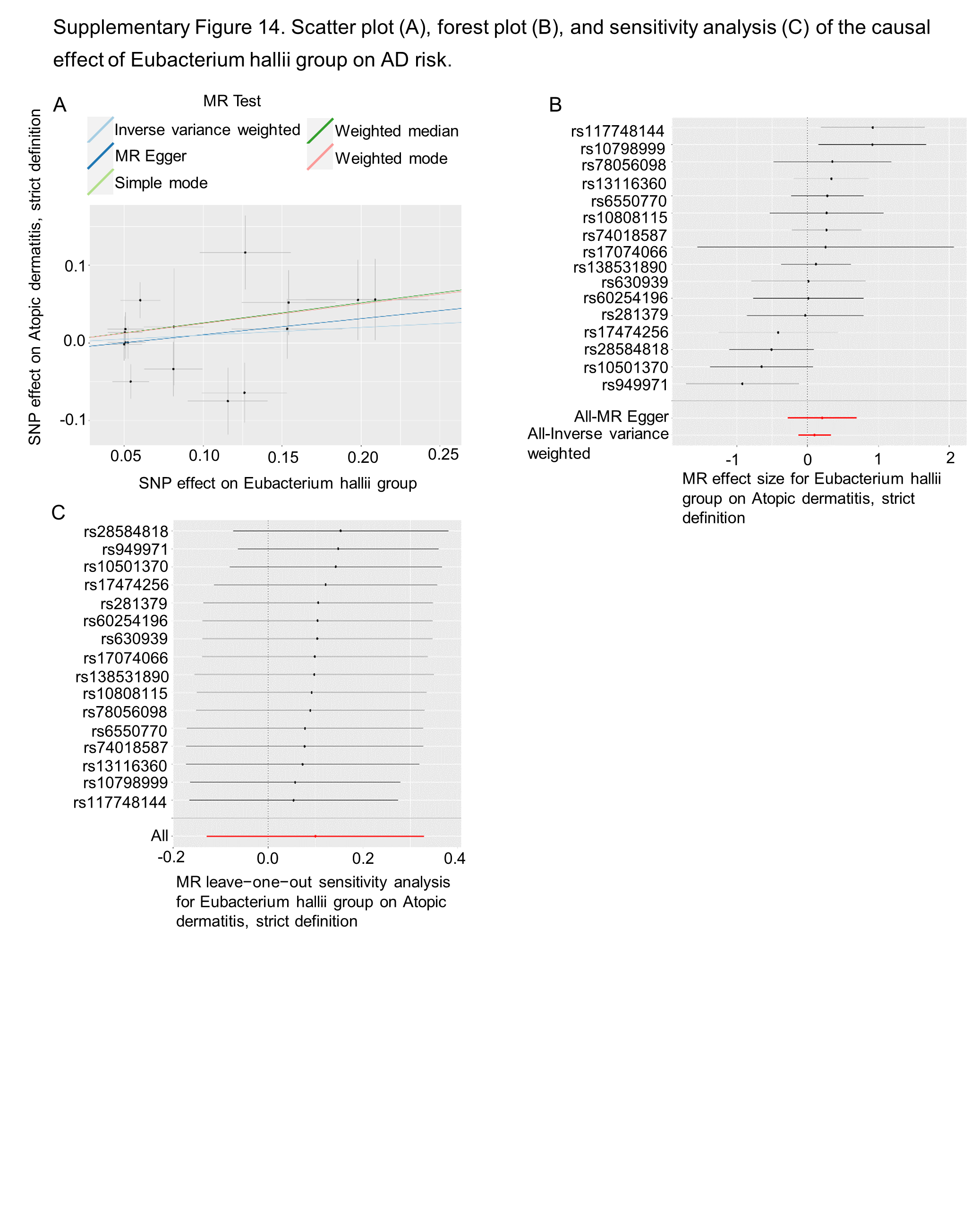

Supplement: Supplementary file 6 [file Data_Sheet_1.ZIP › Supplementary Figure 14.tif]

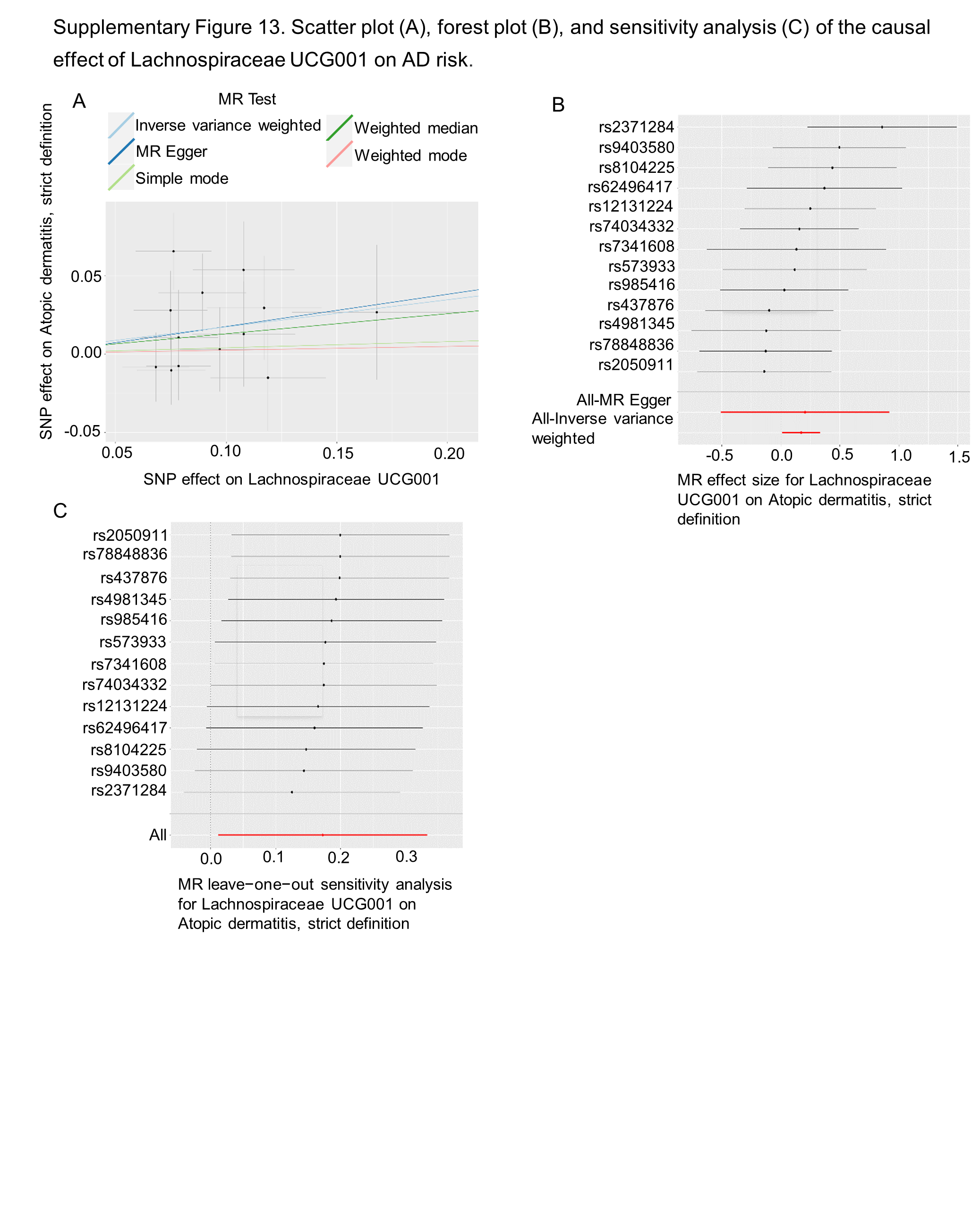

Supplement: Supplementary file 6 [file Data_Sheet_1.ZIP › Supplementary Figure 13.tif]

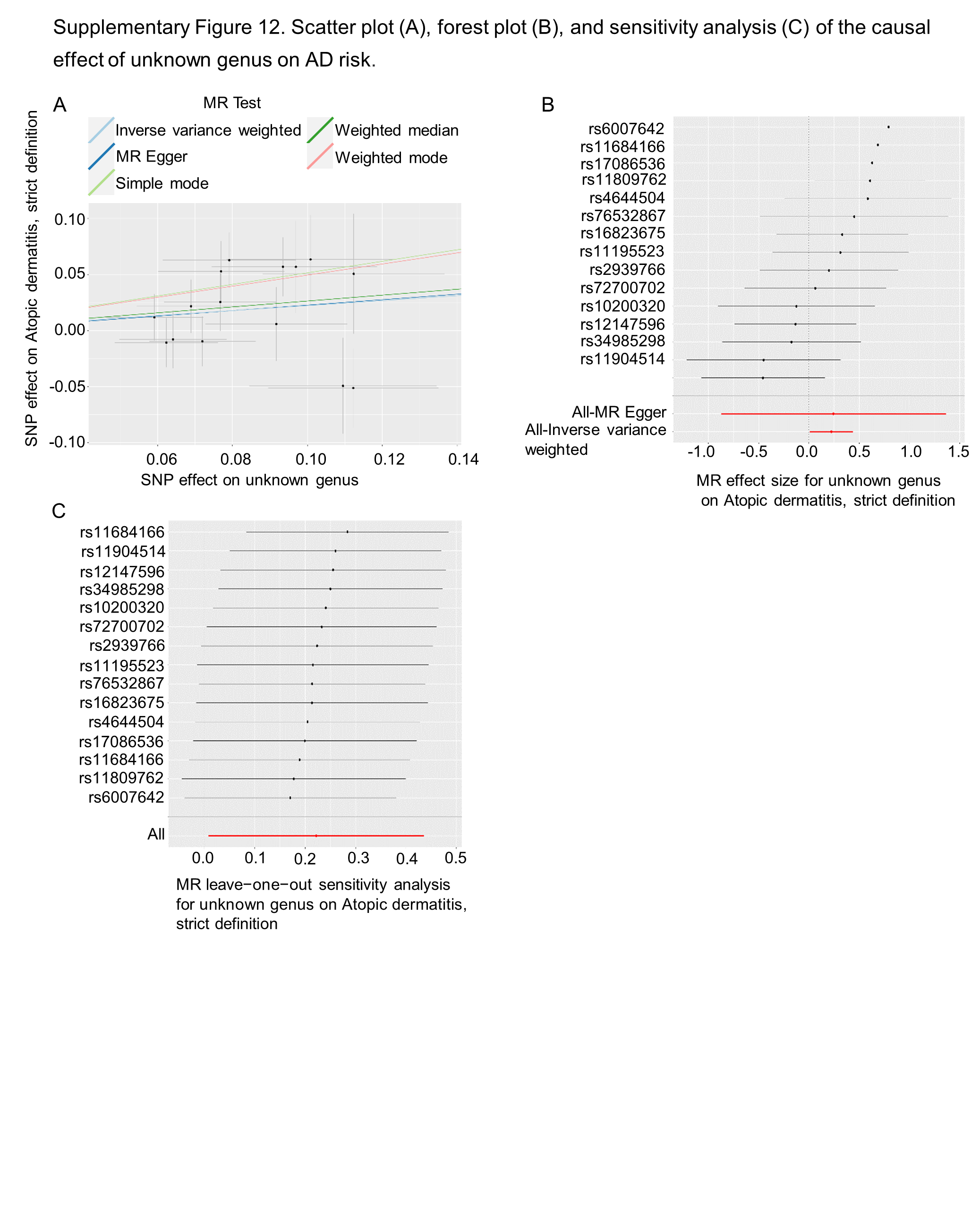

Supplement: Supplementary file 6 [file Data_Sheet_1.ZIP › Supplementary Figure 12.tif]

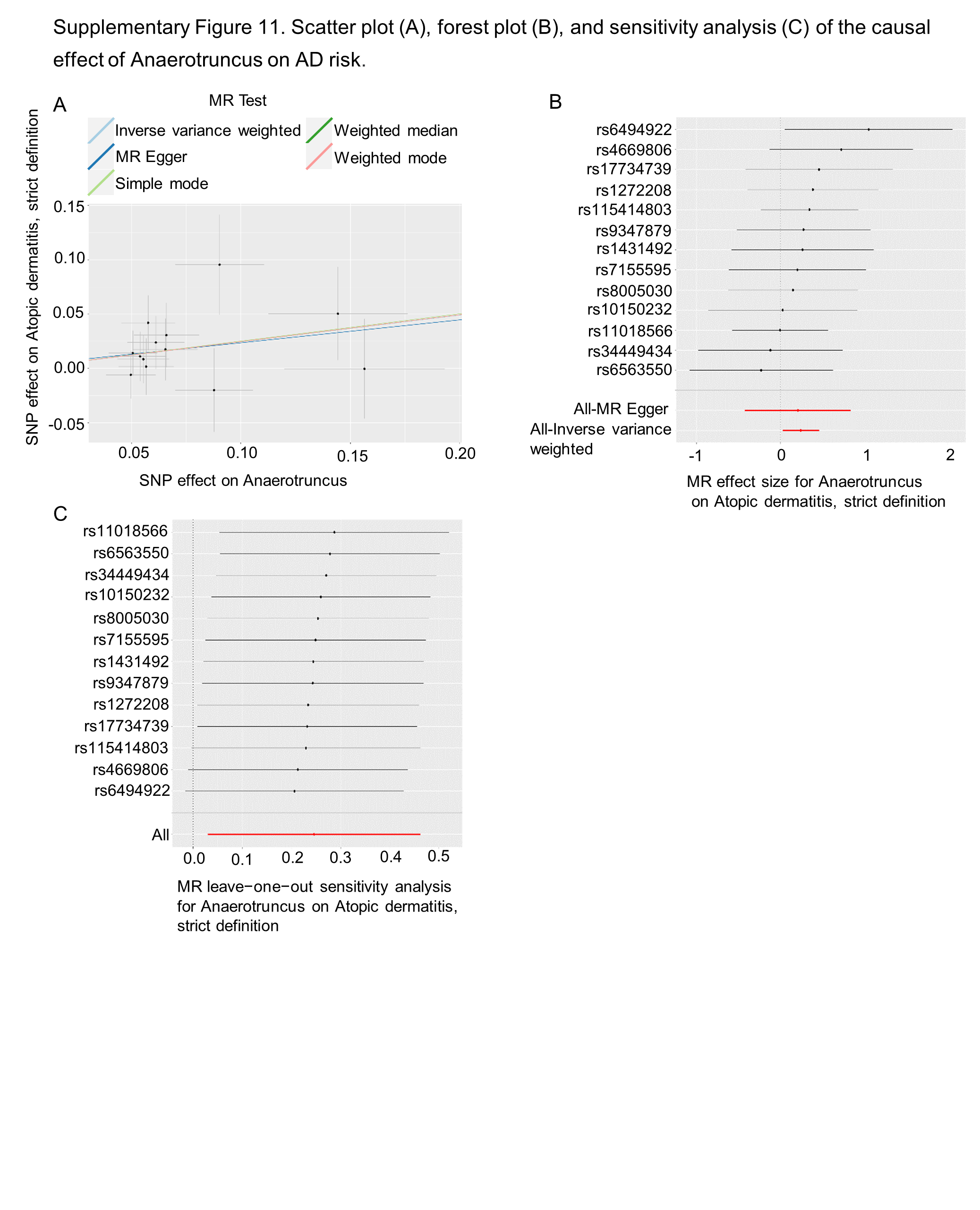

Supplement: Supplementary file 6 [file Data_Sheet_1.ZIP › Supplementary Figure 11.tif]

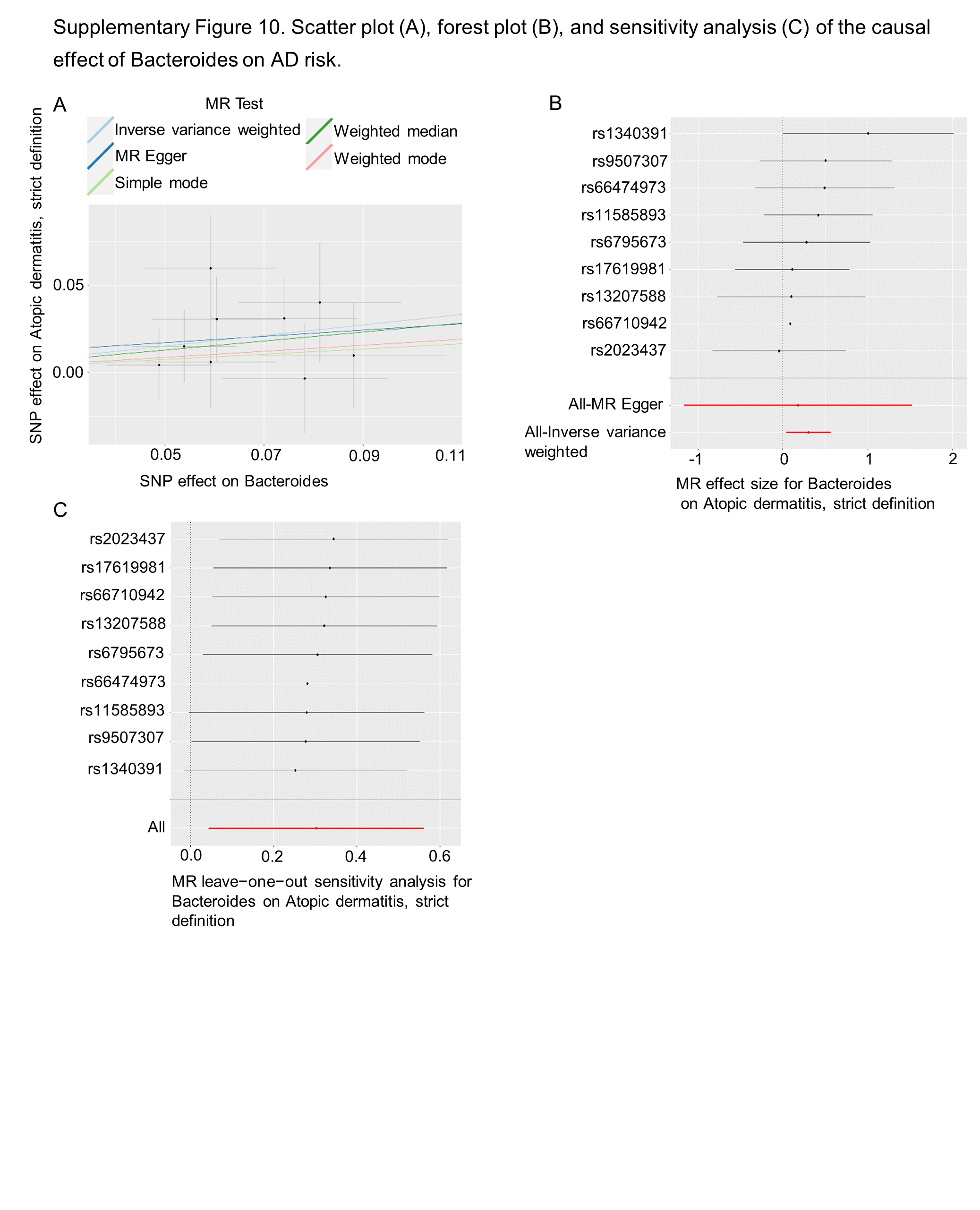

Supplement: Supplementary file 6 [file Data_Sheet_1.ZIP › Supplementary Figure 10.tif]

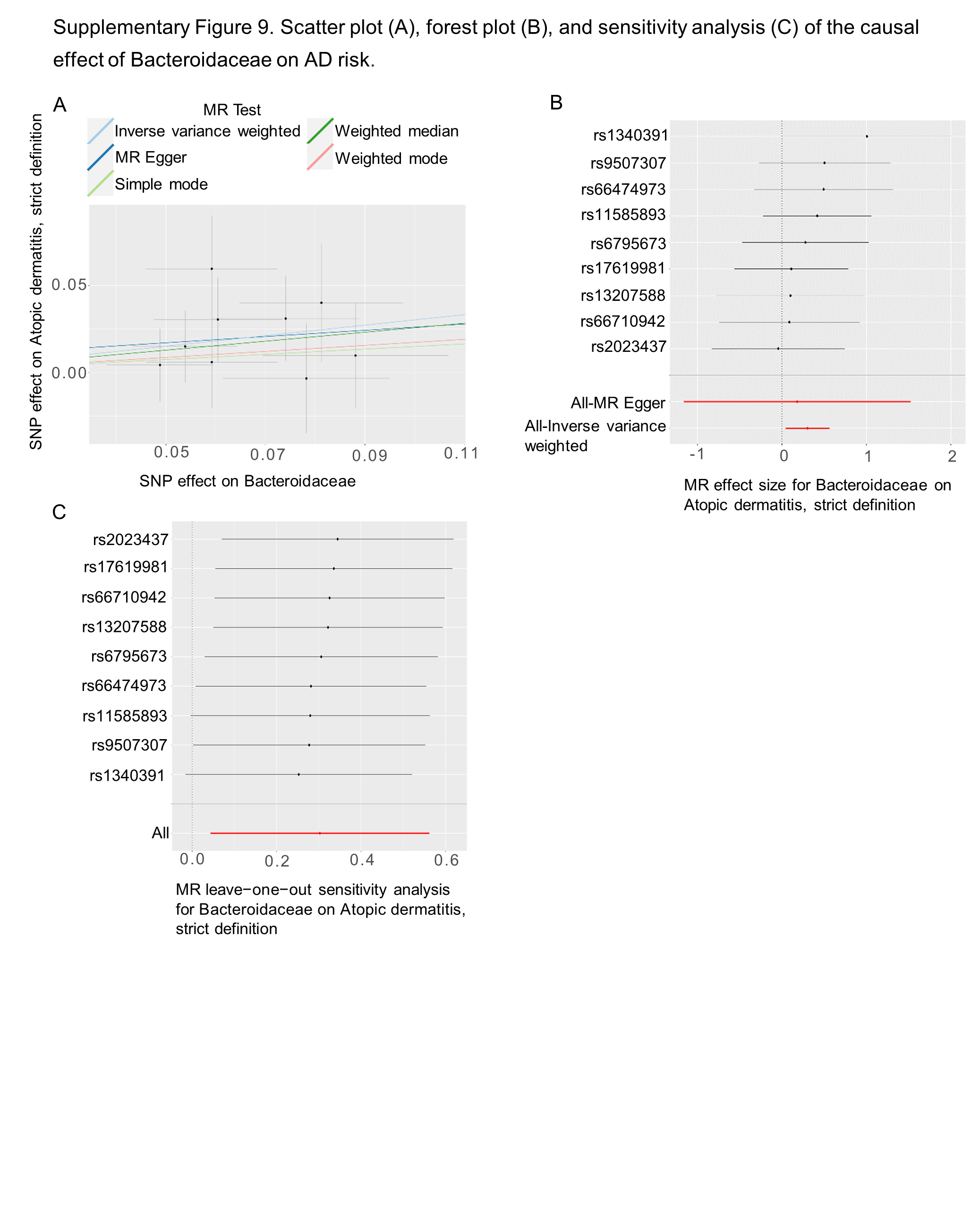

Supplement: Supplementary file 6 [file Data_Sheet_1.ZIP › Supplementary Figure 9.tif]

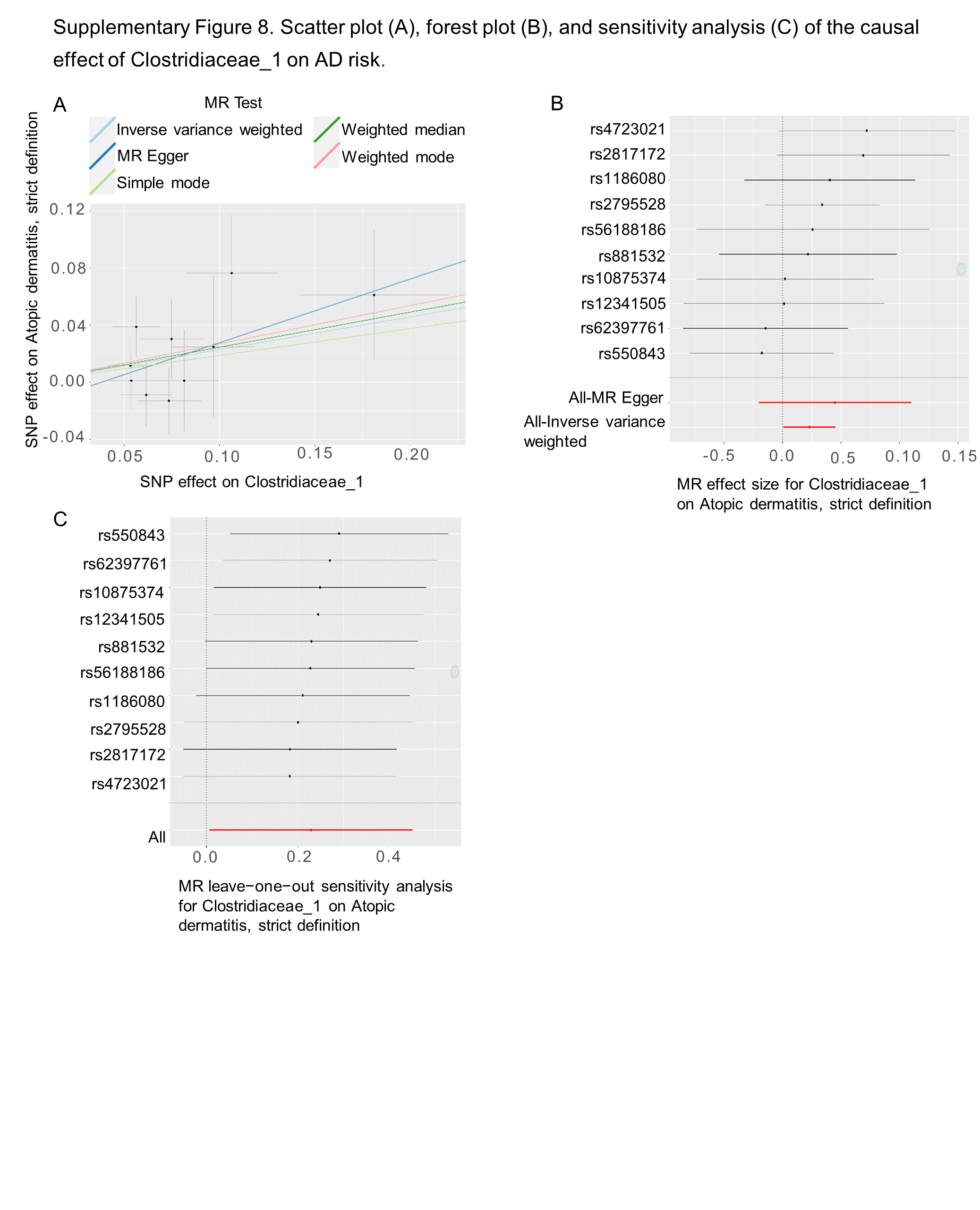

Supplement: Supplementary file 6 [file Data_Sheet_1.ZIP › Supplementary Figure 8.tif]

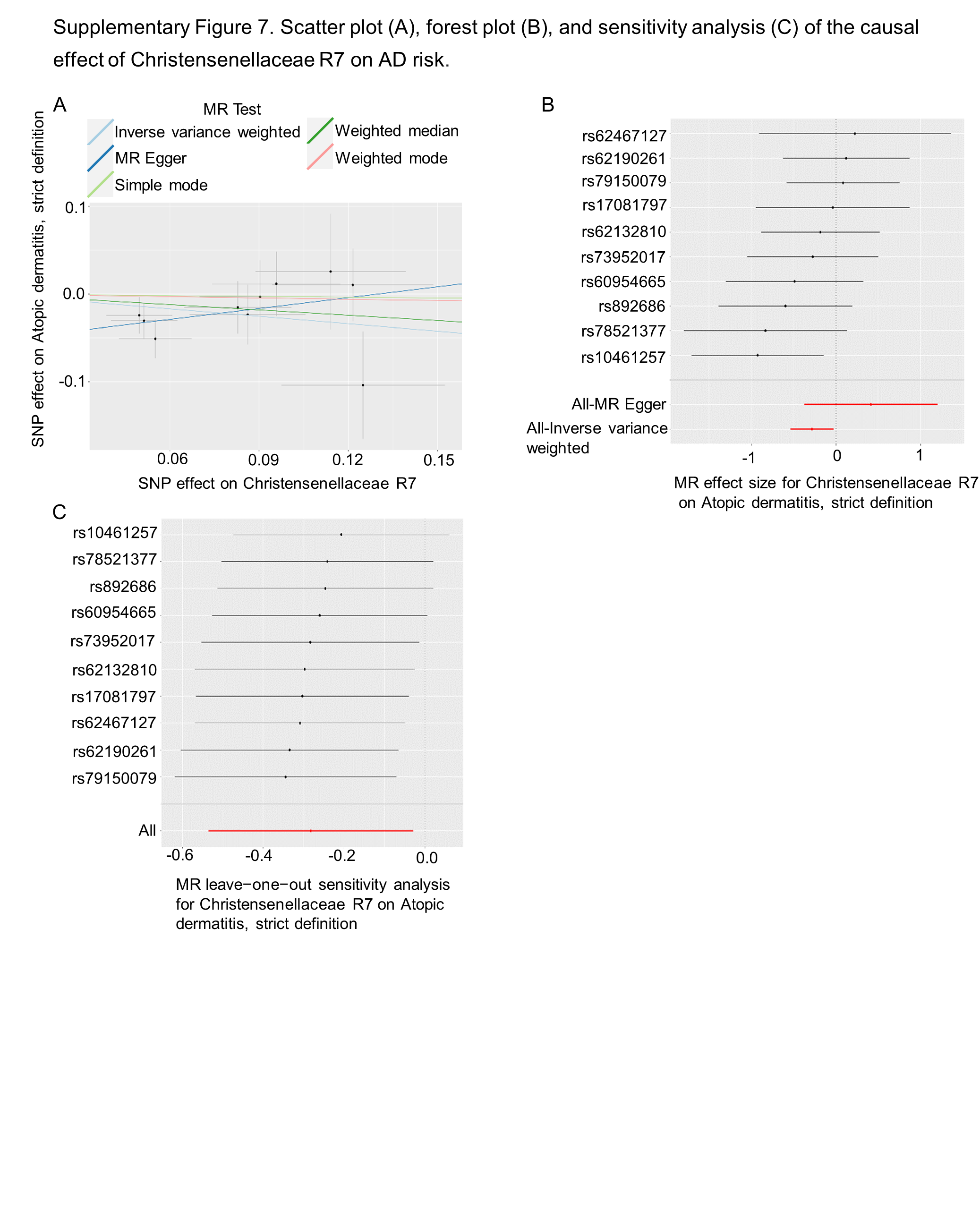

Supplement: Supplementary file 6 [file Data_Sheet_1.ZIP › Supplementary Figure 7.tif]

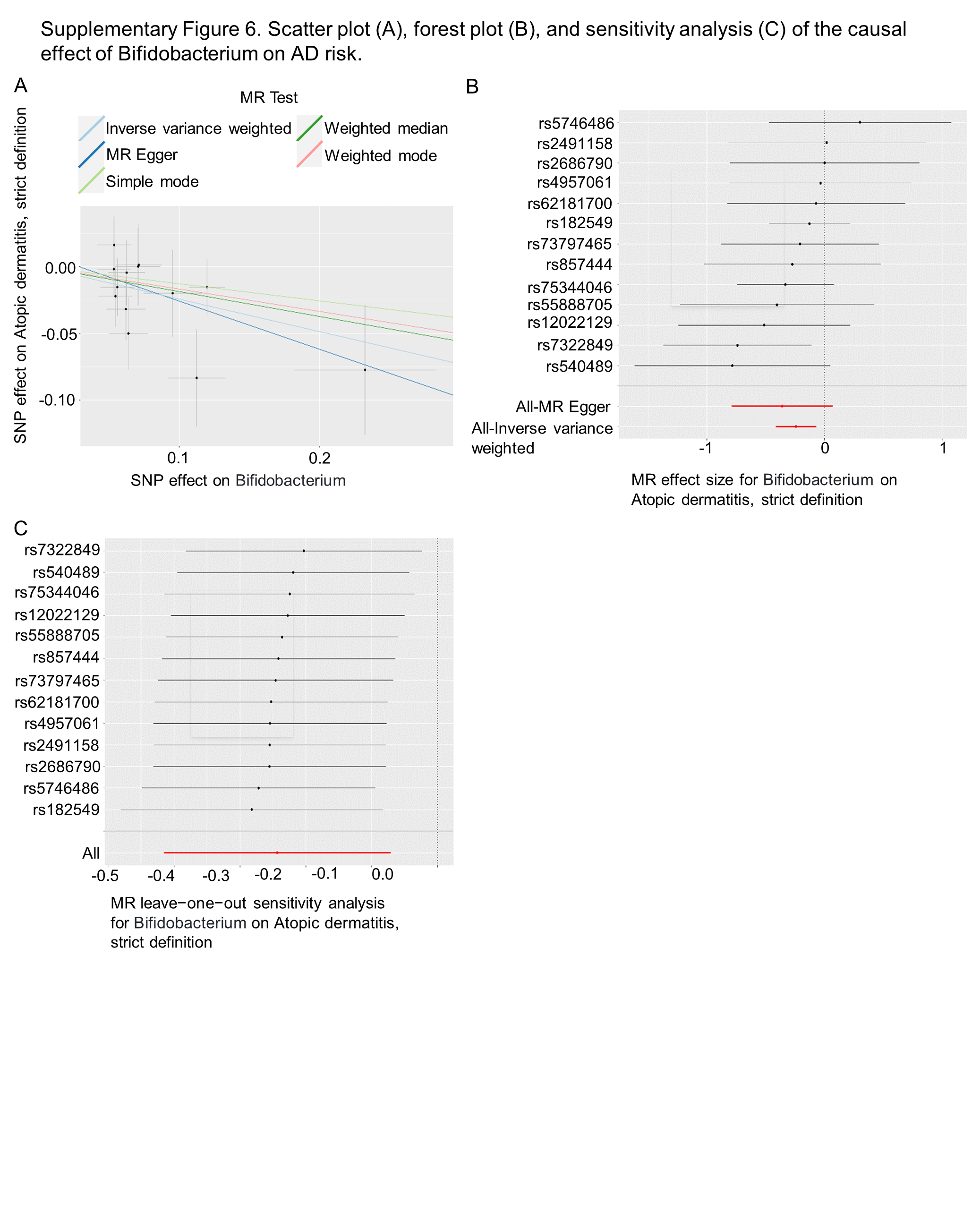

Supplement: Supplementary file 6 [file Data_Sheet_1.ZIP › Supplementary Figure 6.tif]

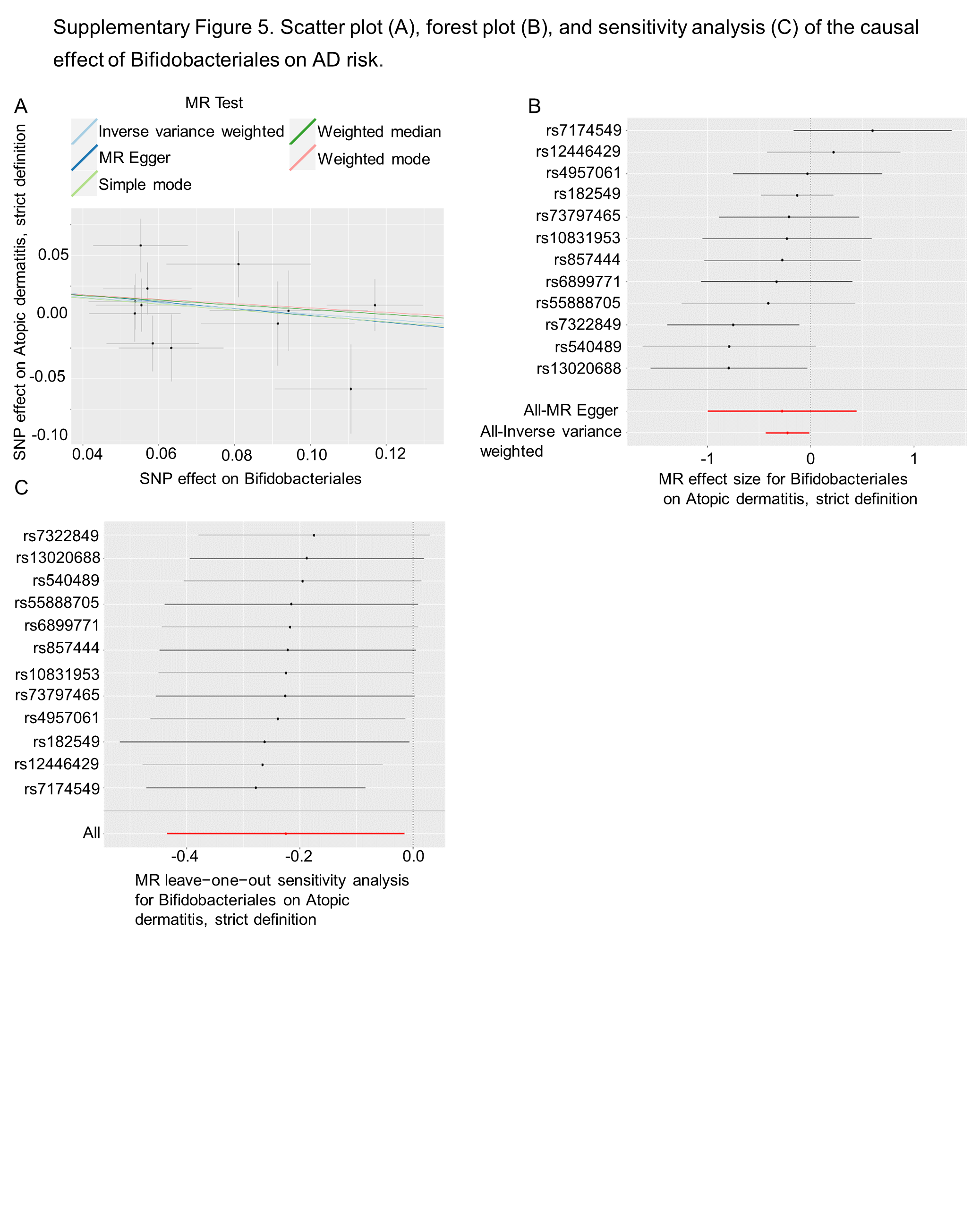

Supplement: Supplementary file 6 [file Data_Sheet_1.ZIP › Supplementary Figure 5.tif]

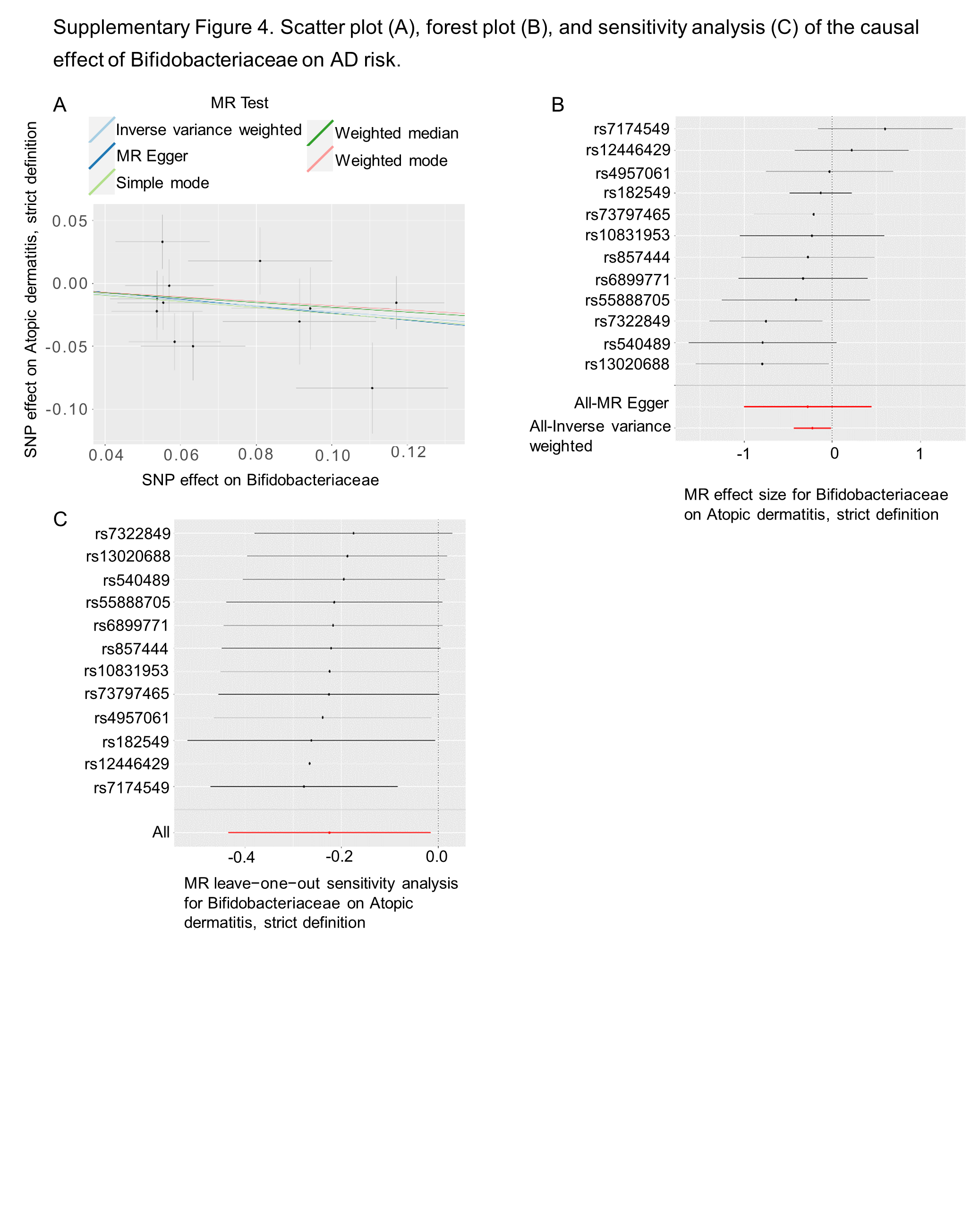

Supplement: Supplementary file 6 [file Data_Sheet_1.ZIP › Supplementary Figure 4.tif]

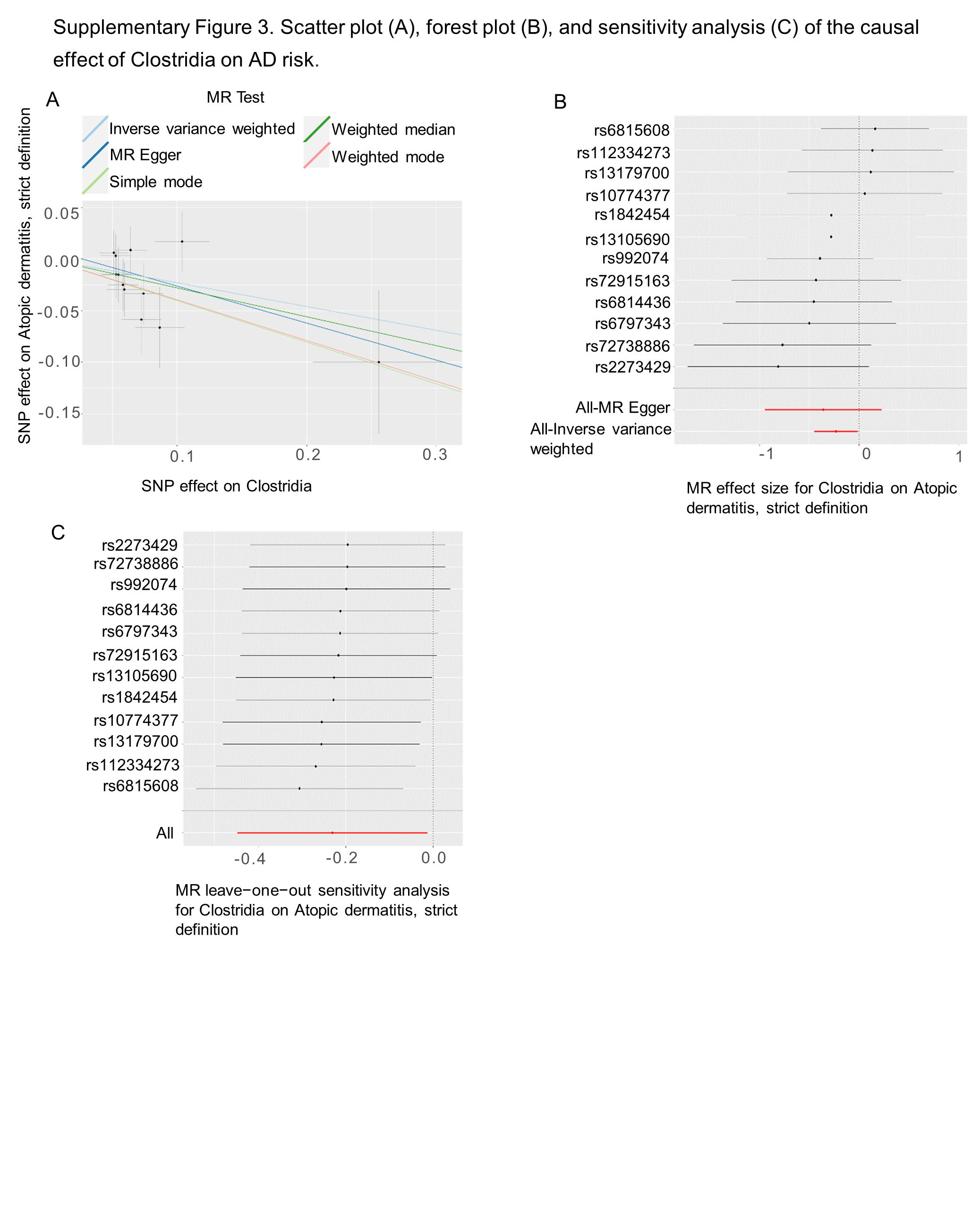

Supplement: Supplementary file 6 [file Data_Sheet_1.ZIP › Supplementary Figure 3.tif]

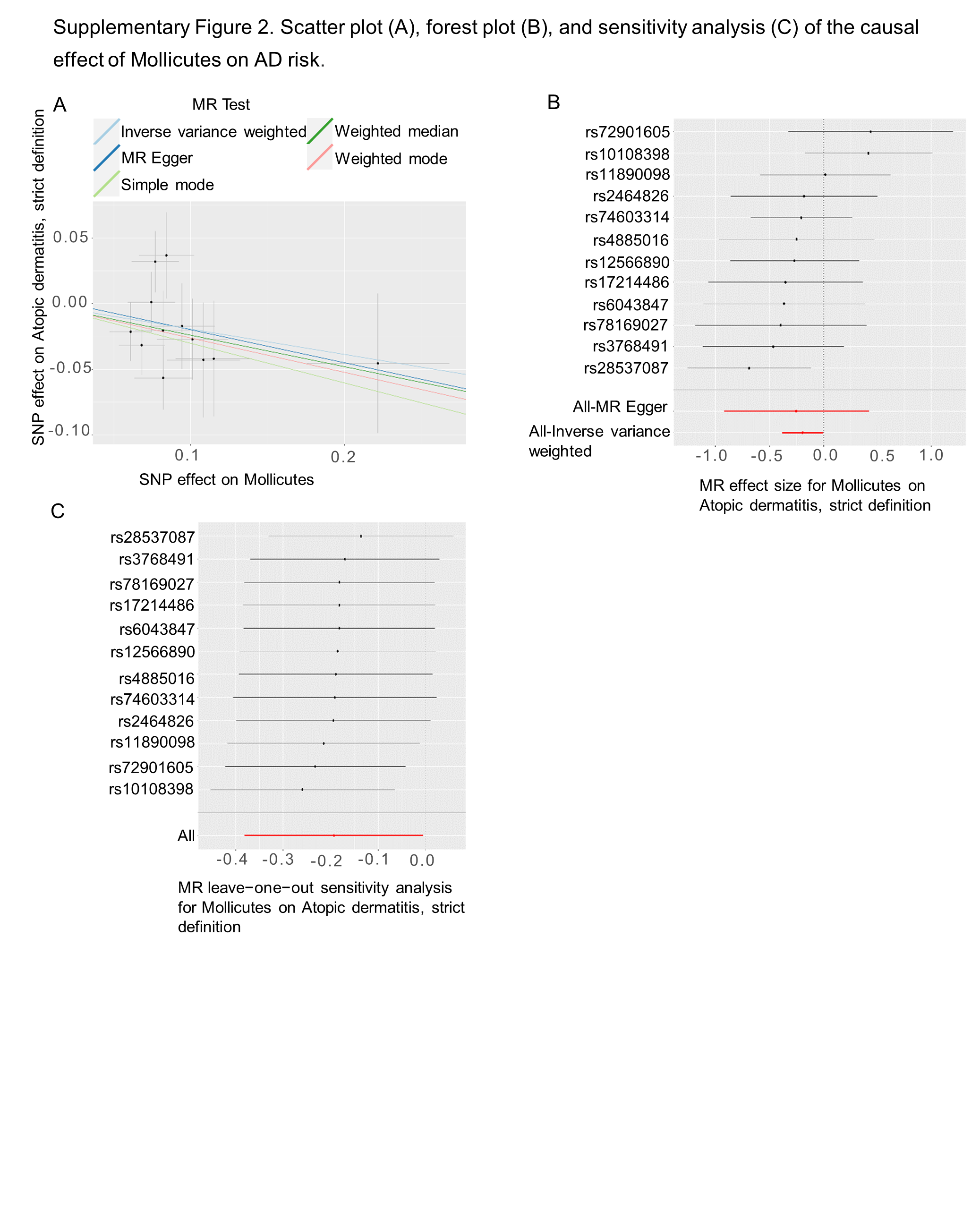

Supplement: Supplementary file 6 [file Data_Sheet_1.ZIP › Supplementary Figure 2.tif]

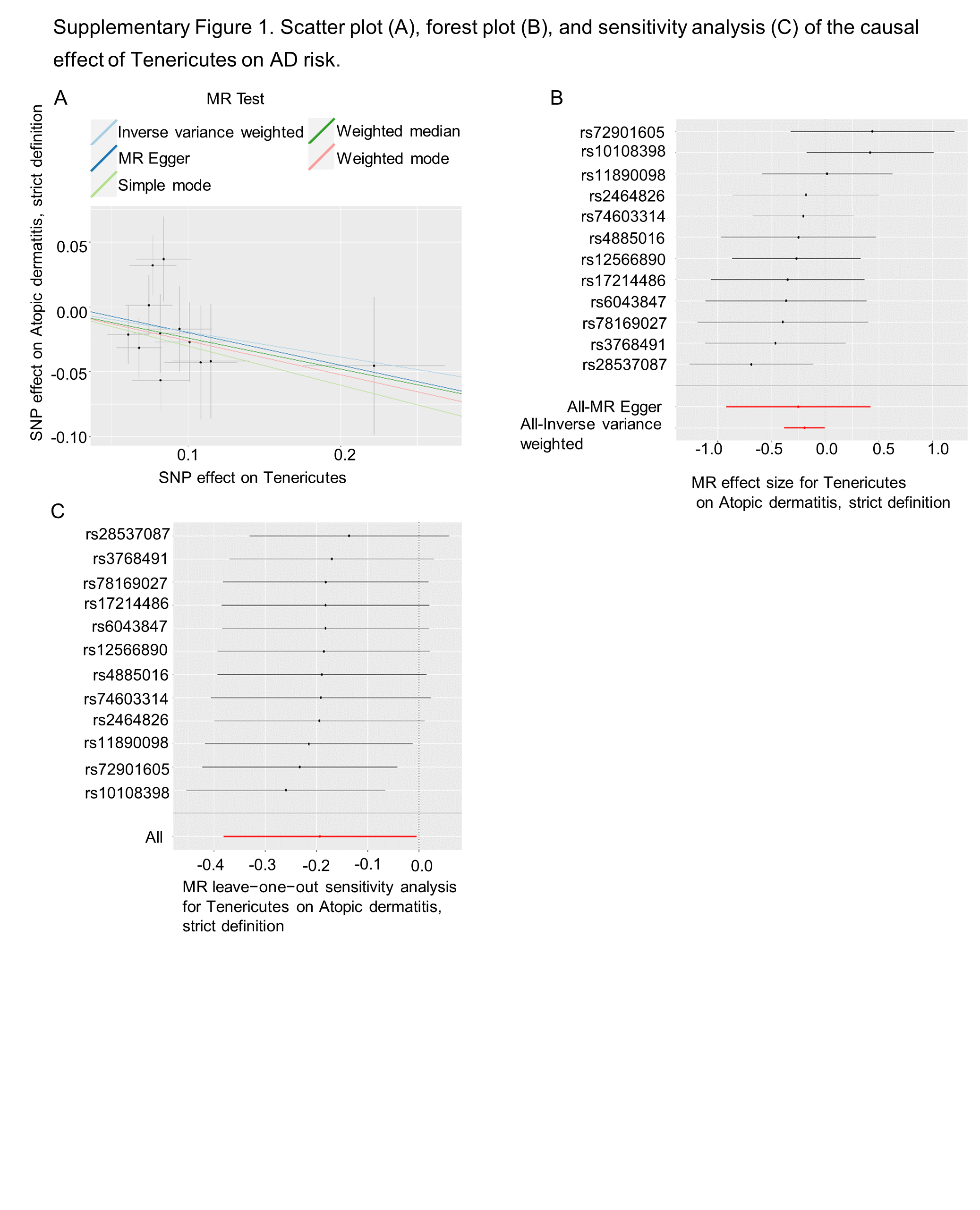

Supplement: Supplementary file 6 [file Data_Sheet_1.ZIP › Supplementary Figure 1.tif]
